# PAK2 promotes CTC cluster formation by phosphorylating E-cadherin to enhance cell-cell adhesion in breast cancer

**DOI:** 10.1186/s13058-025-02199-z

**Published:** 2025-12-21

**Authors:** Lihuang Guo, Jiancheng Li, Wenxing Zhu, Zhuo Wang, Youyou Huang, Zhengyang Sun, Yue Huang, Keqian Xu

**Affiliations:** 1 Department of Laboratory Medicine, The Third Xiangya Hospital, Central South University, Changsha, China; 2 Department of Laboratory Medicine, Xiangya School of Medicine, Central South University, Changsha, China

**Keywords:** Breast cancer, CTC cluster, Metastasis, PAK2, E-cadherin, Cell adhesion

## Abstract

**Background:**

Circulating tumor cell (CTC) clusters exhibit significantly greater metastatic potential than single CTCs and are associated with poorer overall survival in cancers. However, the molecular mechanisms driving CTC cluster formation remain unclear. p21-activated kinase 2 (PAK2) plays a critical role in cytoskeletal remodeling and is frequently associated with advanced tumor progression and poor prognosis. In this study, we explored the role of PAK2 in CTC cluster formation in breast cancer.

**Methods:**

We performed an integrated bioinformatics analysis of transcriptomic profiles from single CTCs and CTC clusters via GEO datasets to identify differentially expressed genes (DEGs) and candidate hub genes associated with CTC clustering. Functional enrichment analyses and gene set enrichment analysis were subsequently conducted to explore relevant pathways. The biological function of the identified hub gene *PAK2* was validated via in vitro CTC cluster formation cell models and in vivo orthotopic in situ breast cancer mouse models. Mechanistic studies focused on PAK2-mediated phosphorylation of E-cadherin. Additionally, the therapeutic potential of targeting PAK2 was evaluated via the use of the selective PAK inhibitor FRAX597 in vivo.

**Results:**

Bioinformatics analyses revealed that CTC clusters are characterized by enhanced cell-cell adhesion, increased proliferative capacity and survival advantages. Among the identified hub genes, *PAK2* was significantly upregulated in breast cancer tissues and cell lines, and its elevated expression was associated with poor patient prognosis. Functional experiments demonstrated that PAK2 promotes CTC cluster formation by increasing E-cadherin phosphorylation at Ser840, thereby strengthening cell-cell adhesion. Pharmacologic inhibition of PAK2 with FRAX597 impaired CTC cluster formation, suppressed tumor growth, reduced metastasis and decreased CTC cluster numbers in vivo.

**Conclusions:**

This study revealed that PAK2 promotes CTC cluster formation and breast cancer metastasis by enhancing E-cadherin-mediated cell-cell adhesion. These results provide novel insights into the molecular mechanisms underlying CTC cluster formation and highlight PAK2 as a potential therapeutic target and diagnostic marker for preventing breast cancer metastasis.

**Supplementary Information:**

The online version contains supplementary material available at 10.1186/s13058-025-02199-z.

## Introduction

Circulating tumor cells (CTCs) are cancer cells that detach from primary or metastatic tumors and enter the bloodstream. They are considered key metastatic precursors across various cancer types and account for approximately 90% of cancer-related deaths, including those from breast cancer [1, 2]. CTCs can be detected in the blood either as single cells or as multicellular aggregates composed of two or more cells, referred to as CTC clusters. Despite comprising only 2–5% of total CTCs, CTC clusters exhibit a 23–50-fold greater metastatic potential than single CTCs, and their presence is strongly associated with poorer overall survival in breast cancer patients [3–5]. Therefore, understanding the mechanisms of CTC cluster formation is critical for developing strategies to prevent cancer metastasis and represents an important area of cancer research.

Previous studies have demonstrated that CTC clusters can arise through intravascular aggregation of single CTCs, with strong cell-cell adhesion playing a pivotal role in this process. This adhesion is characterized by the overexpression of molecules involved in desmosomes and focal junctions [3, 6, 7]. And factors such as CD44 or ICAM1 have also been implicated in mediating cell adhesion and promoting intravascular aggregation of tumor cells [8, 9]. These findings highlight the central role of cell adhesion-related molecules in CTC cluster biology formation. However, the molecular mechanisms underlying this process remain incompletely understood.

p21-activated kinase 2 (PAK2), a member of the p21-activated kinase family, is a serine/threonine kinase activated by Cdc42. Functionally, PAK2 plays a critical role in cytoskeletal remodeling and regulates key biological processes such as cell migration, apoptosis and cell division [10]. In various cancers, high PAK2 expression is associated with tumor progression, metastasis and poor prognosis [11–13]. E-cadherin (encoded by *CDH1*) is a key component of adherens junctions, mediating calcium-dependent cell-cell adhesion and connecting to the actin cytoskeleton via cadherin-catenin complexes [14]. Increasing evidence indicates that PAK2 plays a key role in regulating cell-cell adhesion. PAK2 localizes to adherens junctions and is activated by mechanical tension at E-cadherin adhesion sites, promoting epithelial survival and preventing anoikis, thereby linking PAK2 activity to E-cadherin-mediated adhesion [15, 16]. However, the role of PAK2 in CTC clustering has not yet been explored. In this study, we performed a comprehensive bioinformatics analysis of publicly available transcriptomic datasets from single CTCs and CTC clusters and identified differentially expressed genes (DEGs) and enriched pathways. Among the candidate hub genes, *PAK2* emerged as a potential regulator of CTC cluster formation.

Here, we combined transcriptomic data mining with in vitro and in vivo experiments to elucidate the role of PAK2 in CTC cluster biology. Our findings demonstrate that PAK2 promotes CTC cluster formation by phosphorylating E-cadherin at Ser840 through its kinase activity, thereby enhancing metastatic potential. Elevated PAK2 expression also associated with poor prognosis in breast cancer patients. Furthermore, the PAK2 inhibitor FRAX597 significantly suppressed both CTC cluster formation and lung metastasis in orthotopic breast cancer mouse models. Collectively, this work reveals new insights into the molecular mechanisms driving CTC cluster formation and highlights PAK2 as a potential prognostic biomarker and therapeutic target for preventing metastasis in patients with breast cancer.

## Materials and methods

### Data collection and data preprocessing

RNA-seq datasets of single CTCs and CTC clusters from breast cancer patients were obtained from the GEO database under accession numbers GSE111065 and GSE51827. Specifically, GSE111065 included data from 12 breast cancer patients, comprising 48 single CTCs and 21 CTC clusters, while GSE51827 included transcriptomic profiles from 10 breast cancer patients, comprising 15 single CTCs and 14 CTC clusters. The clinical characteristics of patients in GSE51827 and GSE111065 including the number of patients, age, clinical stage, histological type and the number of single and clustered CTC per patient is provided in Table S1. Raw sequencing data were downloaded and subjected to quality control. Missing values were removed, and probes were mapped to official gene symbols using the annotation files for each dataset platform. For multiple probes corresponding to a single gene, the average expression value was calculated for further analysis.

### Differentially expressed genes analysis

Differential expression analysis between single CTCs and CTC clusters in each dataset was performed using the DESeq2, with method set to median-ratio normalization. DEGs were defined using the following threshold criteria: |log2-fold change| > 2 and adjusted *P*-value < 0.05. Volcano plots were generated via ggplot2 to visualize the DEGs distribution. Common DEGs (coDEGs) between the two datasets were identified using an online Venn diagram tool (http://bioinformatics.psb.ugent.be/webtools/Venn/). GO enrichment analysis (biological process, cellular component, and molecular function) and KEGG pathway analysis of coDEGs were performed using the DAVID database [17]. Enriched terms with adjusted P-value < 0.05 were considered significant and visualized using ggplot2. PPI networks of coDEGs were also constructed using the STRING database [18], visualized in Cytoscape (version 3.10.3) and hub genes were identified using the cytoHubba plugin (top 30 ranked by MCC_SCORE). PAK2 expression was further examined using immunohistochemical (IHC) data from the Human Protein Atlas, and its association with distant metastasis-free survival (DMFS) in breast cancer patients was evaluated using the Kaplan-Meier Plotter tool [19]. Gene set enrichment analysis (GSEA) was performed to investigate the pathways associated with PAK2 expression in single CTCs and CTC clusters. Samples from GSE111065 and GSE51827 were independently divided into high-PAK2 and low-PAK2 groups based on the median expression of PAK2 within each dataset, and GSEA was conducted separately for each dataset using KEGG gene sets (c2.cp.kegg_legacy.v2025.1.Hs.symbols.gmt) in GSEA software (version 4.4.0). Enrichment was considered statistically significant when FDR q-value < 0.25, according to standard GSEA recommendations.

### Cell culture

The human breast cancer cell lines BT549, MDA-MB-231, SKBR3, AU565, along with the normal mammary epithelial cell line MCF10A, were purchased from the American Type Culture Library (ATCC, Manassas, Virginia, USA). All the cells were maintained in the appropriate medium recommended by the suppliers, supplemented with 10% FBS, in a humidified incubator at 37 °C with 5% CO_2_.

### Antibodies, reagents and inhibitors

Antibodies against E-cadherin (Cat#20874-1-AP), Vimentin (Cat#10366-1-AP), Twist (Cat#25465-1-AP) and GAPDH (Cat#10494-1-AP) were purchased from ProteinTech Group (Wuhan, China). Antibodies against PAK2 (Cat# 222584), phospho-PAK2 (S141) (Cat#690384) and phospho-E-cadherin (S840) (Cat# R26278) were purchased from Zen-Bioscience (Chengdu, China). The Cell Counting Kit-8 (CCK-8), Co-IP assay kit, and Hematoxylin and Eosin (H&E) Staining Kits were purchased from Beyotime Institute of Biotechnology (Shanghai, China). The PAK2 inhibitor FRAX597 was purchased from TargetMol (Shanghai, China).

### In vitro model systems for cell cluster formation

The aggregation of breast cancer cells in circulation was mimicked via suspension culture methods, as described by Alexander [20]. A 2 mL cell suspension containing 40,000 cancer cells was seeded per well in a 6-well ultralow attachment U-bottom plate (Corning, USA), and incubated at 37 °C with 5% CO_2_. The cell clusters formed in the 6-well plate were imaged with a Carl Zeiss microscope after 12, 24 and 48 h.

### Quantification of single and clustered CTCs

The quantification of clustered versus single CTCs was performed following previously published methods with minor modifications [6–8]. Briefly, CTCs were imaged under a 10× objective using a standard light microscope. For each sample, twenty randomly selected fields were captured. Single CTCs and CTC clusters (aggregates of two or more cells) were quantified using semi-automated image analysis in ImageJ, followed by manual verification to ensure accuracy. All counting was performed in a blinded manner by two independent researchers. The percentage of clustered CTCs was calculated as the number of clusters divided by the total number of CTC events (single plus clustered) across all analyzed fields.

### Gene overexpression and Silencing experiments

Stable PAK2-overexpressing breast cancer cell lines were generated using lentivirus-mediated transduction (3×Flag-pLenti-GIII-CMV vector from Applied Biological Materials, Vancouver, Canada). The lentiviral control vector (lenti-Con) served as a negative control. BT549 cells were seeded into a 24-well plate (at 0.5 × 10^5^ cells/well). The next day, BT549 cells were infected with lenti-PAK2 or lenti-Con at a multiplicity of infection of 100 for 24 h. Subsequently, the culture medium was replaced and the stable cell lines were screened with 0.5 mg/mL puromycin (Biosharp, Shanghai, China) for 4 weeks and used for subsequent experiments.

RNA interference was performed with Lipo8000 according to the manufacturer’s instructions (Beyotime Institute of Biotechnology, Shanghai, China). The short interfering RNA (siRNA) sequences were synthesized by Sangon (Shanghai, China).

The siRNA sequences used were as follows: siPAK2-1: 5′-AGAAGGA ACTGATCA TTAA-3′; siPAK2-2: 5′-GAAACTGGCCAAACCGTTATT-3′; and NC: 5′-UUCUCCGA ACGUGUCACGUTT-3′.

### Protein isolation and Western blotting

Total protein was extracted from the BT549 cell line via RIPA lysis buffer supplemented with 1% phosphatase inhibitor and 1% protease inhibitor, and the protein concentration was assessed using the BCA protein assay reagent (Beyotime Institute of Biotechnology, Shanghai, China). The protein samples were separated by electrophoresis via 7.5% SDS-PAGE, transferred to a PVDF membrane, and blocked with 5% BSA for 2 h. Then, the membrane was incubated with the following primary.

antibodies for overnight at 4 °C: PAK2 at a dilution of 1:5000, E-cadherin at a dilution of 1:20000, p-PAK2 (S141) at a dilution of 1:2000, p-E-cadherin (S840) at a dilution of 1:2000, and GAPDH at a dilution of 1:20000. The following day, the membranes were washed 3 times with PBST and incubated with their corresponding secondary antibodies at 1:10000 for 1 h. Protein bands were detected by ECL and quantified using ImageJ. For each sample, the intensity of each protein band was first normalized to GAPDH. Phosphorylation levels were then calculated by normalizing the phospho-protein intensity to its corresponding total protein (e.g., p-E-cadherin/total E-cadherin, p-PAK2/ total PAK2). The resulting normalized values were further expressed relative to the SKBR3 cells (for PAK2 analyses) or the corresponding experimental control group, which was set to 1. Data are presented as mean ± SD from from three repeated experiments.

### Protein–protein docking

Protein-protein docking between PAK2 and E-cadherin was performed via the GRAMM docking program. Protein sequences were retrieved from UniProtKB on the basis of gene names, and the optimal protein structures were predicted using SWISS-MODEL. The resulting PDB files were then used as input for the docking. A total of 10 docking models were obtained. Among these, the top-ranked model represented the optimal binding conformation.

### Immunofluorescence microscopy

BT549 cells were seeded on glass-bottom 24-well plates. The cells were fixed with pre-chilled methanol for 10 min, permeabilized with 0.2% Triton X-100 for 5 min, blocked with 5% BSA for 30 min at room temperature, and incubated with primary antibodies overnight at 4 °C, followed by fluorophore-conjugated secondary antibodies. PAK2 was visualized via mouse PAK2 (1:1000, Zen-Bioscience, Cat# 222584, clonality: 8P3-A6-J6 ) followed by a Cy3-conjugated goat anti-mouse secondary antibody and E-cadherin was detected via rabbit E-cadherin (1:800, Proteintech, Cat# 20874-1-AP, clonality: polyclonal) followed by a CoraLite Plus 488-conjugated goat anti-rabbit secondary antibody. Nuclear staining was performed with DAPI and the images were acquired by fluorescence microscopy.

### Coimmunoprecipitation (Co-IP) assay

The interaction between PAK2 and E-cadherin was assessed via a Co-IP assay kit (Beyotime Institute of Biotechnology) according to the manufacturer’s protocol. First, the BT549 cells were lysed. Then, 500 µL of cell lysates containing 20 µL of Protein A + G magnetic beads were incubated with antibodies against PAK2 (1/2000) or IgG as the negative control, and a certain proportion of the supernatant without any antibody was used as the positive control (Input), followed by incubation for 2 h, with tumbling, at room temperature. The magnetic beads were separated via magnetic force and then washed with ice-cold lysis buffer. Finally, the magnetic beads were immersed in SDS-PAGE sample loading buffer and boiled for 5 min at 95 °C. Following magnetic separation for 10 s, western blotting was performed to visualize the immunoprecipitated samples.

### Colony formation, wound healing assay and transwell assays

For the colony formation assays, 1000 untreated and transfected BT549 cells were seeded in 6-well plates/well, and the medium was changed every two days. After 12 days, the medium was discarded, and the colonies were fixed with 4% PFA. Then the cells were stained with 0.5% crystal violet (Solarbio, Beijing, China) for 30 min and rinsed three times with PBS. Colonies were imaged and counted using ImageJ.

For the wound healing assay, untreated and transfected BT549 cells at a density of a total of 5 × 10^6^ cells/well were inoculated into a 6‑well plate and incubated at 37 °C for 24 h until a cell monolayer was created when the cells reached 70–80% confluence. Subsequently, a 200‑µL pipette tip was used to create a straight scratch in the middle of the cell monolayer and washed with PBS. Following culturing with serum‑free medium for 48 h at 37 °C, images of the migrated cells at 0, 24 and 48 h.

were captured using an inverted light microscope (magnification, ×10; Carl Zeiss) and quantified using ImageJ.

For the transwell assay, 2 × 10^5^ cells untreated or transfected BT549 cells were seeded onto membrane inserts with 8 μm size in 24-well plates (LABSELECT, China) which precoated with Matrigel for the invasion assay. The lower chamber was filled with RPMI 1640 supplemented with 20% FBS. Following incubation for 24 h at 37 °C, the cells on the lower surface of the membrane were fixed and stained via the methods described for the colony formation assay. The invasive cells were quantified using an inverted light microscope and quantified using ImageJ.

### Orthotopic breast cancer in BALB/c-nu mice and CTC analyses

All the mouse experiments were approved by the Institutional Animal Care and Use Committee (IACUC) of Central South University (approved number: CSU-2025-0127). The in vivo orthotopic breast cancer model employed in this study was based on the protocol previously described by Conner et al. [21]. BT549 cells were trypsinized, suspended in PBS and then mixed with Matrigel matrix (Corning, USA) at a ratio of 1:1 to achieve a final concentration of 1.0 × 10^5^/µL, and the cell suspension was kept on ice until injection. At 4–6 weeks of age, 12 female BALB/c-nu mice were injected with 2 million cells into the fourth right mammary fat pad via a 25G needle. After one week, the mice were randomly divided into two groups: the control group, which received an intraperitoneal (i.p.) injection of saline every other day, and the FRAX597-treated group, which received 3 mg/kg i.p. every other day. The mice were monitored for tumor growth for about 3 weeks. The tumor burden was monitored using digital calipers every three days. After 3 weeks or until the maximum tumor burden of 1.0 cm^3^ was reached or significant ulceration occurred, the mice were sacrificed. Blood samples were collected via cardiac puncture, and lungs were collected for further H&E staining. The histologic analysis of H&E-staining lungs was performed to quantify metastases in accordance with previously established methodologies [21–23]. Briefly, the largest cross-sectional area of each lung was selected and scanned at high resolution (20× objective) using a digital slide scanner. The images were exported as TIFF files and imported into ImageJ. The pixel scale was calibrated using the embedded scale bar (2000 μm in this study). To facilitate lesion identification, images were converted to 8-bit grayscale, and the “Threshold” function was applied to distinguish metastatic foci. For counting the number of metastatic foci, the “Analyze/Particles” function was applied to the thresholded image to automatically count discrete metastatic lesions. For measuring lesion size, each lesion was manually outlined using the “Freehand Selection” tool, and the lesion area was quantified using “Analyze/Measure” function.

For the CTC analysis in this model, the ISET technology was used to quantify the number of single CTCs or CTC clusters [24]. In brief, after blood samples were collected from the mice, the RBCs were lysed with red blood cell lysis buffer (Solarbio, China) and filtered through a membrane with 8 μm diameter pores. The CTCs were retained on the membrane, and the leukocytes passed through the pores because CTCs are larger than leukocytes. The CTCs were subsequently identified via immunocytochemical (ICC) staining. The membranes were examined via fluorescence, and the numbers of single CTCs and CTC clusters were counted. Pancytokeratin (Pan-CK) and E-cadherin were detected with primary antibodies followed by incubation with a CoraLite Plus 488-conjugated goat anti-rabbit secondary antibody. Vimentin and Twist were detected with primary antibodies followed by Cy3-conjugated goat anti-mouse secondary antibodies. CD45 was detected via a CoraLite Plus 647-conjugated anti-mouse CD45 antibody. CTCs were identified as pan-CK/E-cadherin + and/or vimentin/Twist + and CD45- with a morphology consistent with that of a nucleated cell. CTC clusters were defined as aggregations of two or more individual CTCs containing distinct nuclei and intact cytoplasmic membranes.

### Statistical analysis

Continuous data are presented as the means ± SDs. Statistical analyses were performed via GraphPad Prism 9.5. Differences between two groups were evaluated via unpaired Student’s t-tests. For comparisons among multiple groups, one-way ANOVA was used to assess statistical significance. Statistical significance was indicated as *P* < 0.05(*), *P* < 0.01(**), *P* < 0.001(***), and *P* < 0.0001(****); “ns” indicates no significant difference. All experiments were repeated at three times.

## Results

### Identification of DEGs associated with CTC clusters compared to single CTCs

**Fig. 1:**
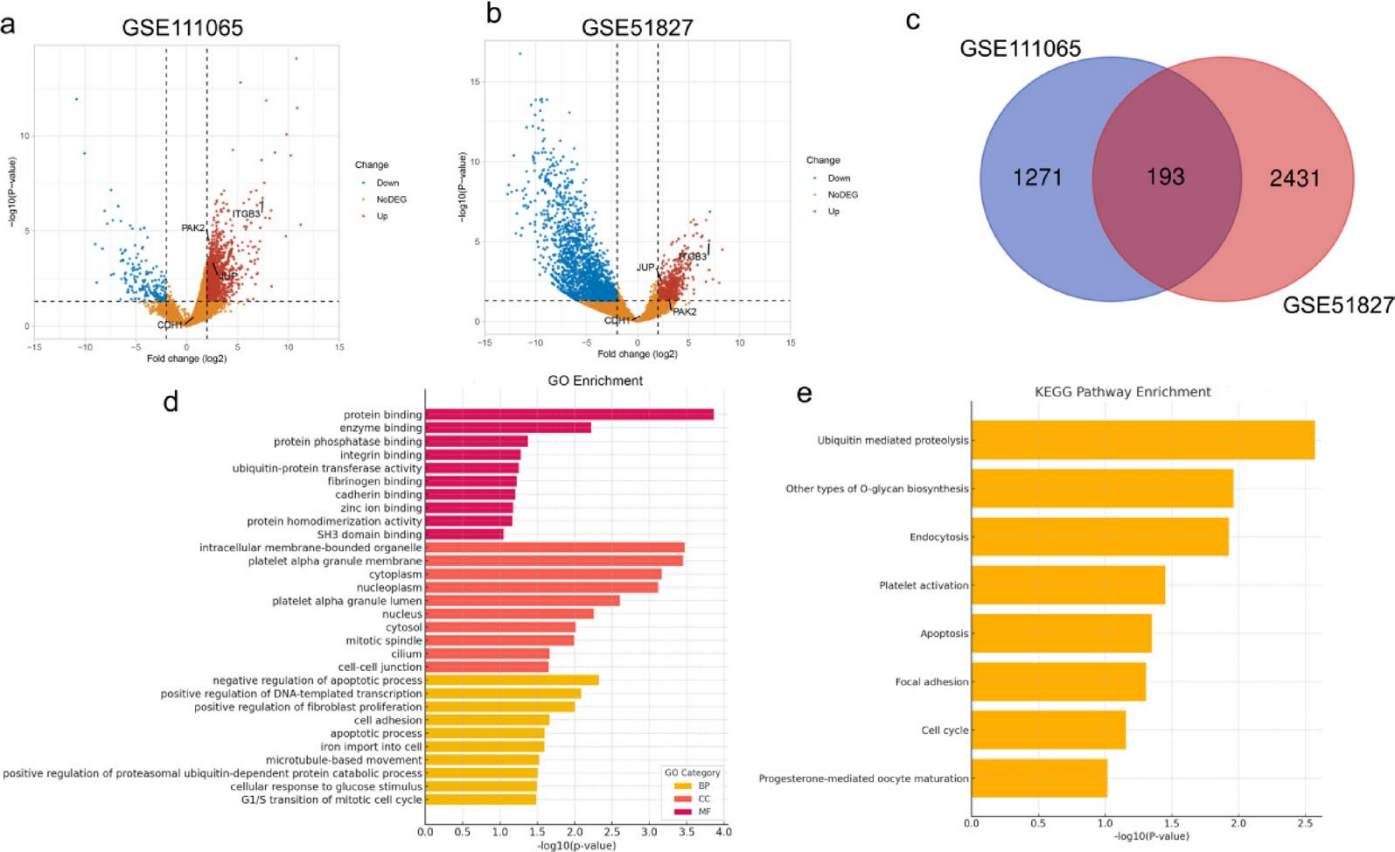
Identification of DEGs between CTC clusters and single CTCs. **a**, **b** Volcano plots of DEGs from the GSE111065 and GSE151827 datasets. Upregulated genes are shown in brown; downregulated genes are shown in blue. **c** Venn diagram showing the overlap of DEGs between the GSE51827 and GSE111065. **d**, **e** GO and KEGG pathway enrichment analyses of the coDEGs

In GSE111065, 1273 genes were upregulated and 191 were downregulated in CTC clusters compared with single CTCs, whereas GSE51827 identified 604 upregulated and 2020 downregulated genes (Fig. 1a, b). A total of 193 coDEGs were shared between the two datasets, including 156 upregulated and 37 downregulated genes (Fig. 1c and Table S2). GO enrichment analysis showed that these coDEGs were involved in biological processes (BP) related to the cell cycle, apoptosis and cell adhesion. The coDEGs were enriched in cellular components (CC) of cell-cell junction, intracellular membrane-bounded organelle and platelet alpha granule membrane. And molecular functions (MF) were enriched in protein binding, integrin binding and cadherin binding (Fig. 1d ). KEGG pathway analysis highlighted roles in cell cycle, apoptosis, ubiquitin-mediated proteolysis, and focal adhesion (Fig. 1e). These findings demonstrated that CTC clusters exhibit enhanced proliferative capacity, survival advantages, and, notably, stronger intercellular adhesion, suggesting that reinforced cell-cell adhesion may be a key mechanism driving CTC cluster formation and contributing critically to their metastatic potential.

### Identification of *PAK2* as the hub gene of CTC cluster formation

**Fig. 2:**
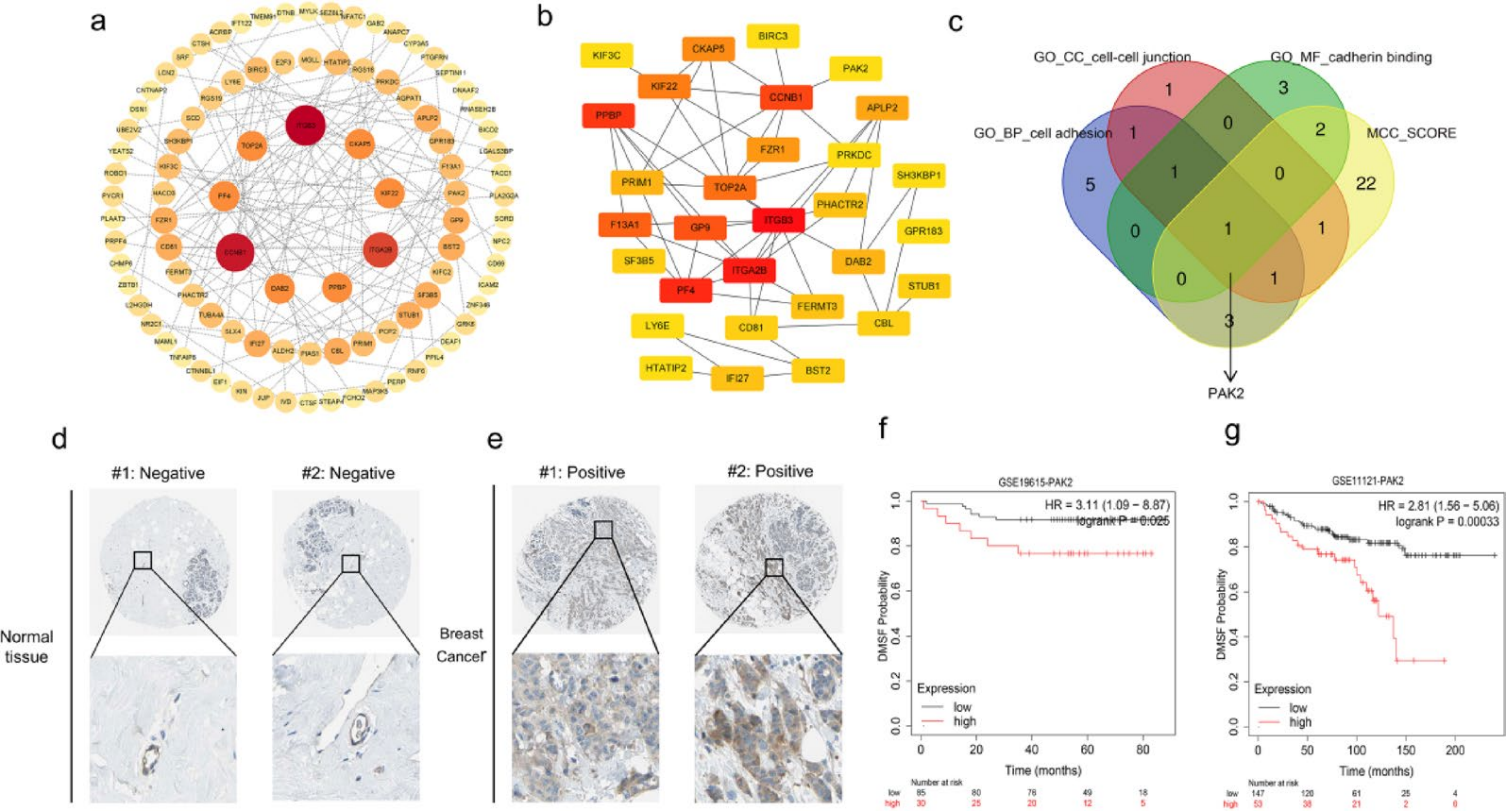
Protein-protein interaction network analysis, hub gene identification, and prognostic significance of PAK2 in breast cancer. **a** PPI network constructed for 193 coDEGs. **b** Top 30 hub genes identified using the CytoHubba plugin in Cytoscape based on MCC scores. **c** Venn diagram highlighting the selection of PAK2 as a hub gene. **d**, **e** Representative IHC staining images of PAK2 expression in normal breast tissue and breast cancer tissues from the Human Protein Atlas database. **f**, **g** Kaplan-Meier analysis of the prognostic impact of PAK2 expression on DMFS in breast cancer patients

A protein-protein interaction network was generated from the 193 coDEGs (Fig. 2a), and the top 30 nodes with the highest maximal clique centrality (MCC) scores were identified (Fig. 2b). In this study, we focused on the genes which involved in cell adhesion that may promote CTC cluster formation. Therefore, *PAK2* was the only gene overlapping between the adhesion-related GO terms (including GO_BP_cell adhesion, GO_CC_cell-cell junction, GO_MF_cadherin binding) and the top 30 genes of MCC-score (Fig. 2c). Detailed information on these genes is provided in Tables S3. In addition, IHC data from the HPA database revealed that PAK2 was typically undetectable in normal breast tissue, but highly expressed in breast cancer samples (Fig. 2d, e). Furthermore, high PAK2 expression correlated with significantly poorer DMFS in breast cancer patients (Fig. 2f, g). Therefore, these results suggest that PAK2 may play an important role in CTC cluster formation in breast cancer and PAK2 was chosen for subsequent analysis and experimental validation.

### High PAK2 expression in breast cancer cell lines is associated with CTC cluster formation

**Fig. 3:**
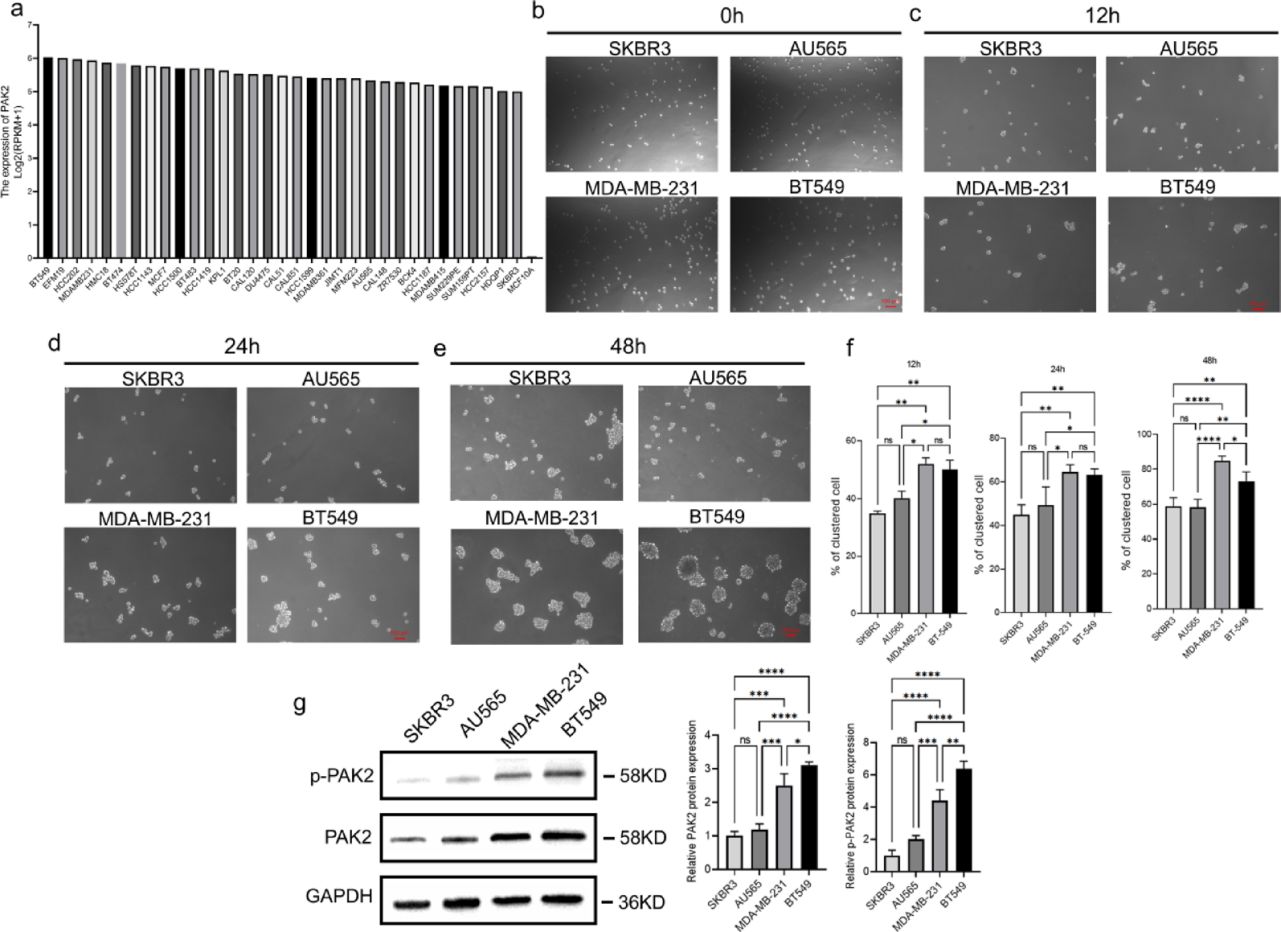
PAK2 expression and cluster formation in breast cancer cell lines. **a** mRNA expression levels of *PAK2* in breast cancer cell lines and the normal mammary epithelial cell line MCF10A. **b**–**f** Quantification of cell cluster formation in four breast cancer cell lines under suspension culture conditions at 12, 24, and 48 h. **g** Immunoblot analysis of total PAK2 and phospho-PAK2 (Ser141) levels in BT549, MDA-MB-231, SKBR3, and AU565 breast cancer cells. SKBR3 cells were used as the reference (set to 1) for relative expression

PAK2 mRNA expression was markedly elevated in breast cancer cell lines compared with normal MCF10A cells, as indicated by the Cancer Cell Line Encyclopedia (CCLE) [25] (Fig. 3a). Given that triple-negative and HER2-positive breast cancer subtypes are highly aggressive and associated with poor prognosis and that CTCs serve as valuable prognostic and predictive biomarkers in these subtypes [26, 27], we selected two triple-negative breast cancer cell lines (BT549, MDA-MB-231) and two HER2-positive breast cancer cell lines (SKBR3, AU565) for subsequent functional studies, with triple-negative cell lines exhibiting the highest aggressiveness. Among these, the HER2-positive SKBR3 cell line was chosen as the reference because it exhibited the lowest PAK2 expression.

Using the in vitro model-systems for cell cluster formation, the BT549 and MDA-MB-231 cells exhibited greater clustering ability than the other cell lines at 12, 24 and 48 h (Fig. 3c, e). Consistently, the BT549 and MDA-MB-231 cell lines also presented elevated PAK2 expression and phosphorylation levels (Fig. 3g). Thus, cell clustering ability was found to be positively associated with the endogenous expression of PAK2. BT549 cells were chosen as the primary cell line for our further experiments, as these cells presented higher levels of both total and phosphorylated PAK2 than MDA-MB-231cells. In subsequent analyses, we also observed a potential association between PAK2 and E-cadherin. Notably, MDA-MB-231 cells lack E-cadherin expression [28], whereas BT549 cells retain E-cadherin expression, making them a more appropriate model for investigating the relationship between PAK2 and E-cadherin.

### PAK2 facilitates cluster formation and regulates the proliferation, migration, and invasion of breast cancer cells

**Fig. 4:**
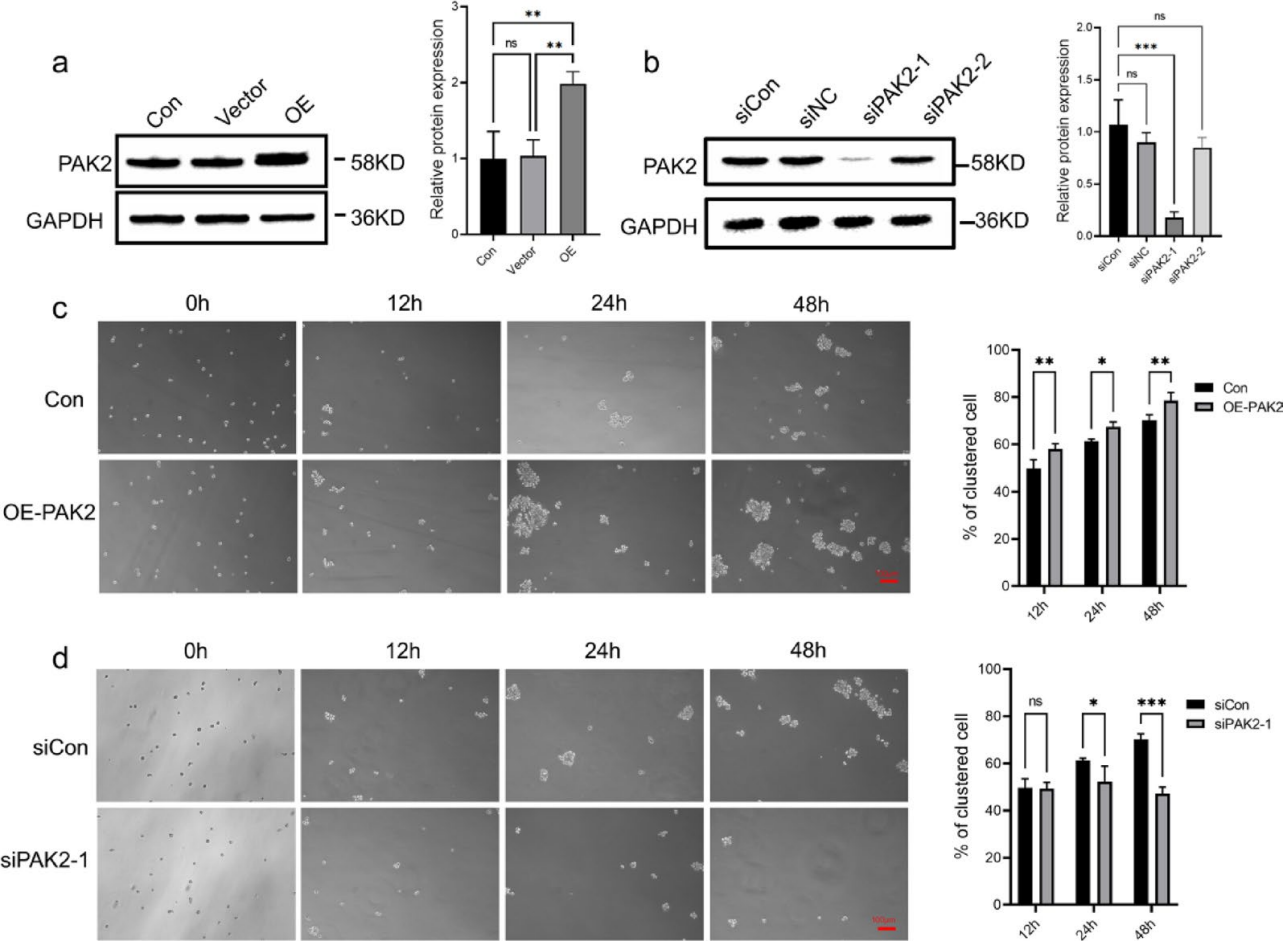
PAK2 promotes cell cluster formation. **a**, **b** Immunoblot analysis showing PAK2 expression in BT549 cells after overexpression or knockdown. **c** Representative images and quantification of cluster formation in BT549 cells transfected with control vector (Con) or lenti-PAK2 (OE-PAK2). **d** Representative images and quantification of cluster formation in BT549 cells transfected with control siRNA (siCon) or siPAK2-1

**Fig. 5:**
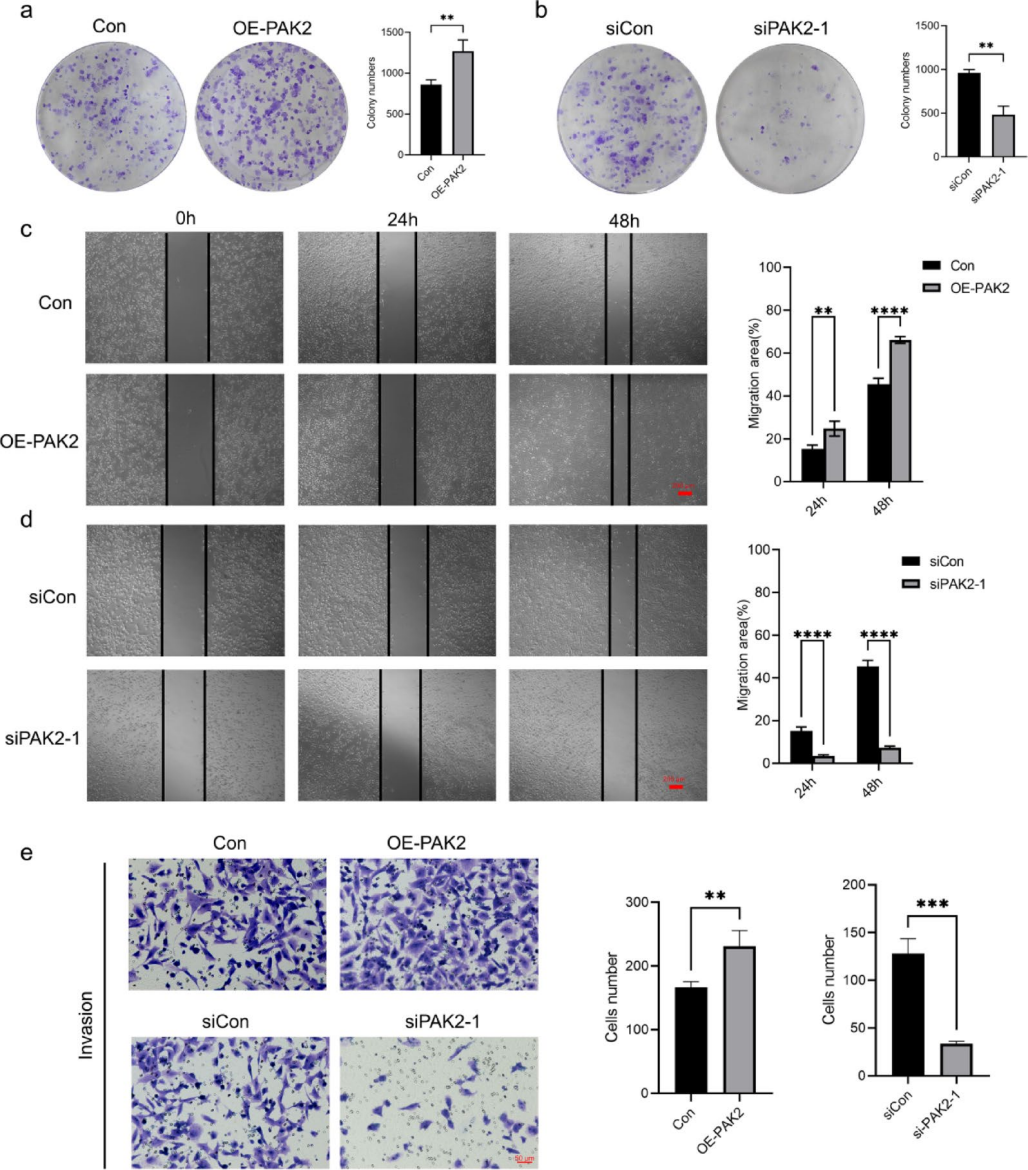
Effects of PAK2 on breast cancer cell progression. **a**, **b** Colony formation assays performed in BT549 cells with PAK2 overexpression or knockdown. **c**, **d** Wound healing assays evaluating the migration of BT549 cells with PAK2 overexpression or knockdown. **e** Transwell assays evaluating the invasive capacity of BT549 cells with PAK2 overexpression or knockdown

To determine whether PAK2 contributes to cell aggregation, we generated BT549 cells with stable PAK2 overexpression (Fig. 4a). The results revealed that BT549 cells overexpressing PAK2 formed a significantly greater number of cell clusters than control cells (Fig. 4c). Conversely, knockdown of PAK2 using two siRNAs, among which siPAK2-1 showed the strongest silencing efficiency (Fig. 4b), significantly reduced cluster formation (Fig. 4d). These findings indicate that PAK2 enhances the clustering ability of breast cancer cells. Additionally, we assessed whether PAK2 also affected other malignant phenotypes of BT549. PAK2 overexpression markedly increased BT549 cell proliferation, whereas PAK2 knockdown suppressed colony growth (Fig. 5a, b). Similarly, PAK2 promoted cell migration and invasion, as evidenced by wound healing and Transwell assays, while PAK2 knockdown impaired both behaviors (Fig. 5c, e). Collectively, these results demonstrate that PAK2 drives CTC cluster formation and regulates malignant behaviors in breast cancer cells.

### PAK2 mediates cell cluster formation via phosphorylation of E-cadherin

**Fig. 6:**
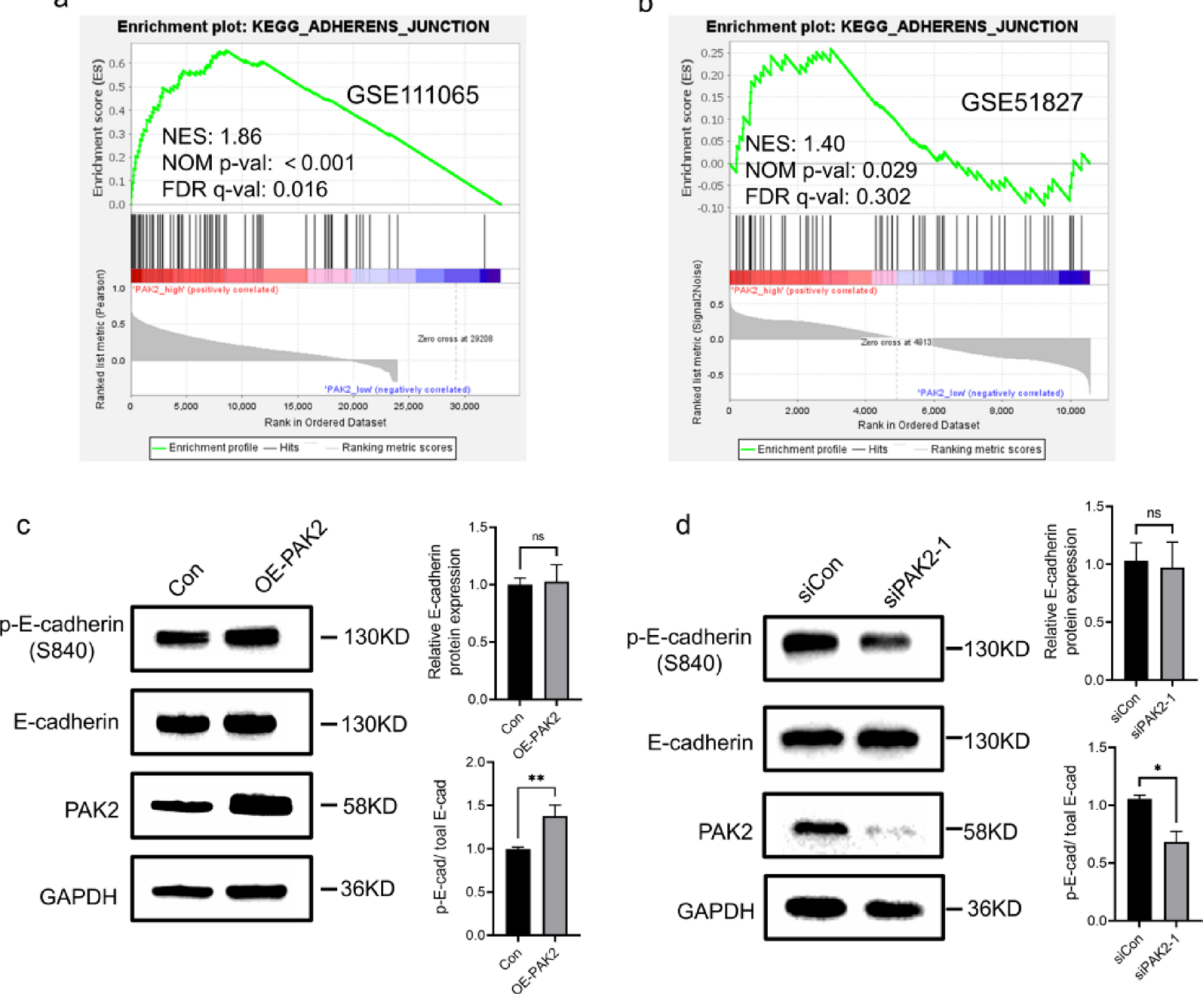
PAK2 mediates cell cluster formation via E-cadherin phosphorylation. **a**, **b** GSEA showing the association between PAK2 and the adherens junction pathway. **c**, **d** Immunoblot analysis of E-cadherin and phosphorylated E-cadherin (Ser840) in BT549 cells after PAK2 overexpression or knockdown

Through GO and KEGG enrichment analysis of the coDEGs of single CTCs and CTC clusters, *PAK2* was shown to increase cell-cell adhesion to promote tumor cell aggregation. GSEA was performed to elucidate the specific underlying molecular mechanisms. In GSE111065, the high-PAK2 group showed significant enrichment of adherens junction pathway (FDR q = 0.016) (Fig. 6a). Although the enrichment did not reach FDR significance in GSE51827 (Fig. 6b), the consistent direction of enrichment across both datasets supports a functional link between PAK2 and adherens junction signaling, suggesting that PAK2 may promote CTC cluster formation by regulating adherens junctions.

Then we investigated the E-cadherin protein expression in both OE-PAK2 and si-PAK2 BT549 cells, and found that PAK2 overexpression or knockdown did not alter E-cadherin protein levels. Given that phosphorylation of the E-cadherin cytoplasmic domain can modulate cell-cell adhesion [29, 30], we next investigated the phosphorylation of the E-cadherin. The results showed that phosphorylation of E-cadherin at Ser840 was significantly increased by PAK2 overexpression and significantly reduced by PAK2 knockdown (Fig. 6c, d). Together, these data demonstrate that PAK2 promotes aggregation through the phosphorylation of E-cadherin, thereby modulating adhesion strength.

### PAK2 binds directly to E-cadherin

**Fig. 7:**
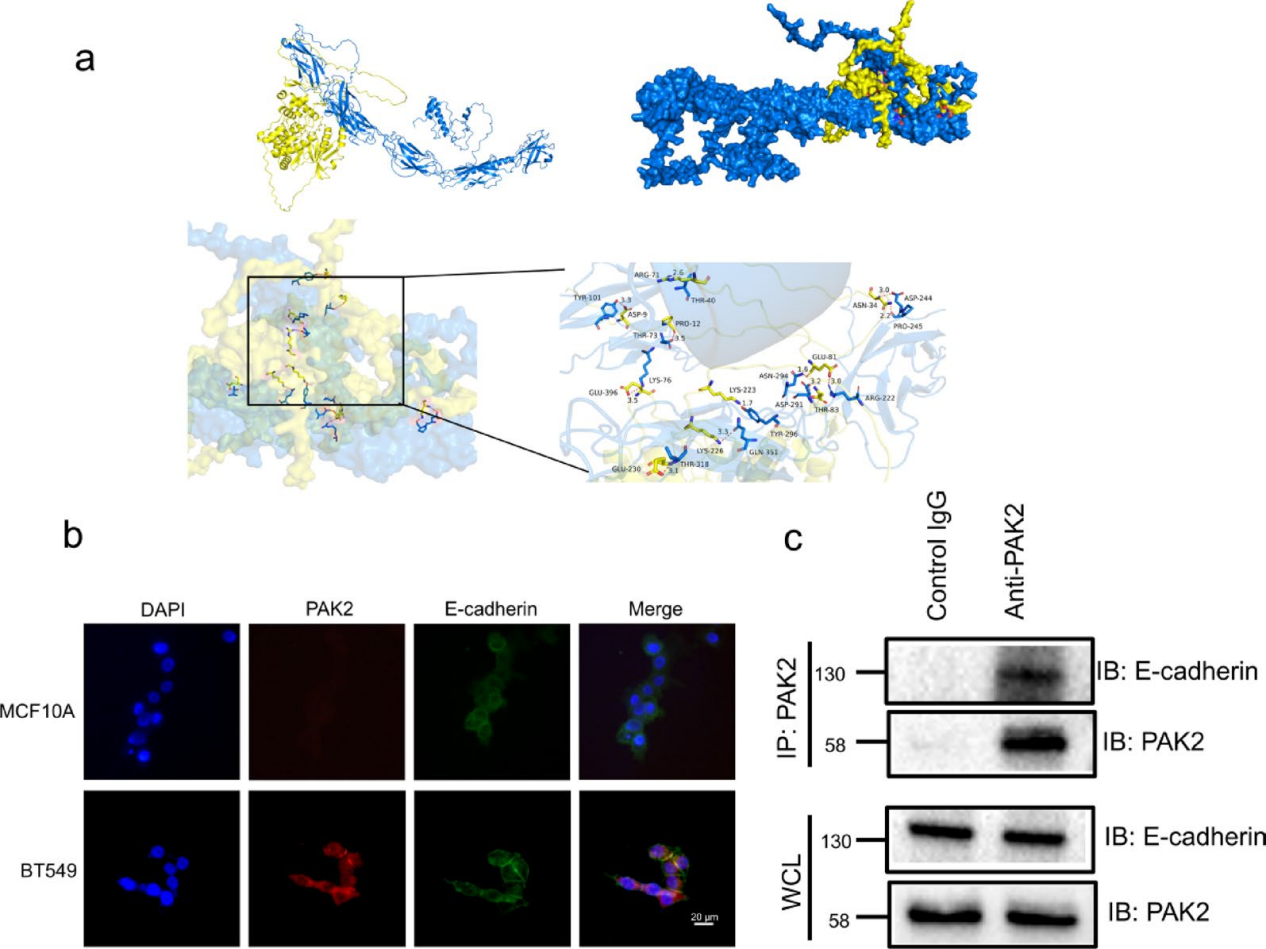
PAK2 interacts and colocalizes with E-cadherin. **a** Protein docking map and binding site of PAK2 for E-cadherin. The yellow color represents PAK2, and the blue color represents E-cadherin. **b** IF staining illustrating the colocalization of PAK2 and E-cadherin in MCF10A and BT549. **c** Co-IP analysis showing the association between PAK2 and E-cadherin in breast cancer cells

To determine whether PAK2 can directly interact with E-cadherin, we first performed protein-protein docking. The docking results revealed the predicted binding interfaces and structural interactions between the two proteins (Fig. 7a). In the structural model, PAK2 is shown in yellow, E-cadherin is shown in blue, and the predicted binding site is highlighted in red. The calculated binding affinity was − 9.4 kcal/mol, indicating a strong and potentially stable interaction, likely mediated by hydrogen bonding. Then we performed IF analysis and found that PAK2 and E-cadherin can colocalize in breast cancer cells (Fig. 7b). MCF10A cells used as a control exhibited strong membrane-localized E-cadherin staining, and the low PAK2 signal observed in MCF10A was in line with our results (Fig. 7b). Co-IP further demonstrated a physical interaction between PAK2 and E-cadherin in BT549 cells (Fig. 7c). Together, our data show that PAK2 binds directly to E-cadherin to promote the formation of CTC clusters.

### The PAK2 inhibitor FRAX597 decreases cell aggregation

**Fig. 8:**
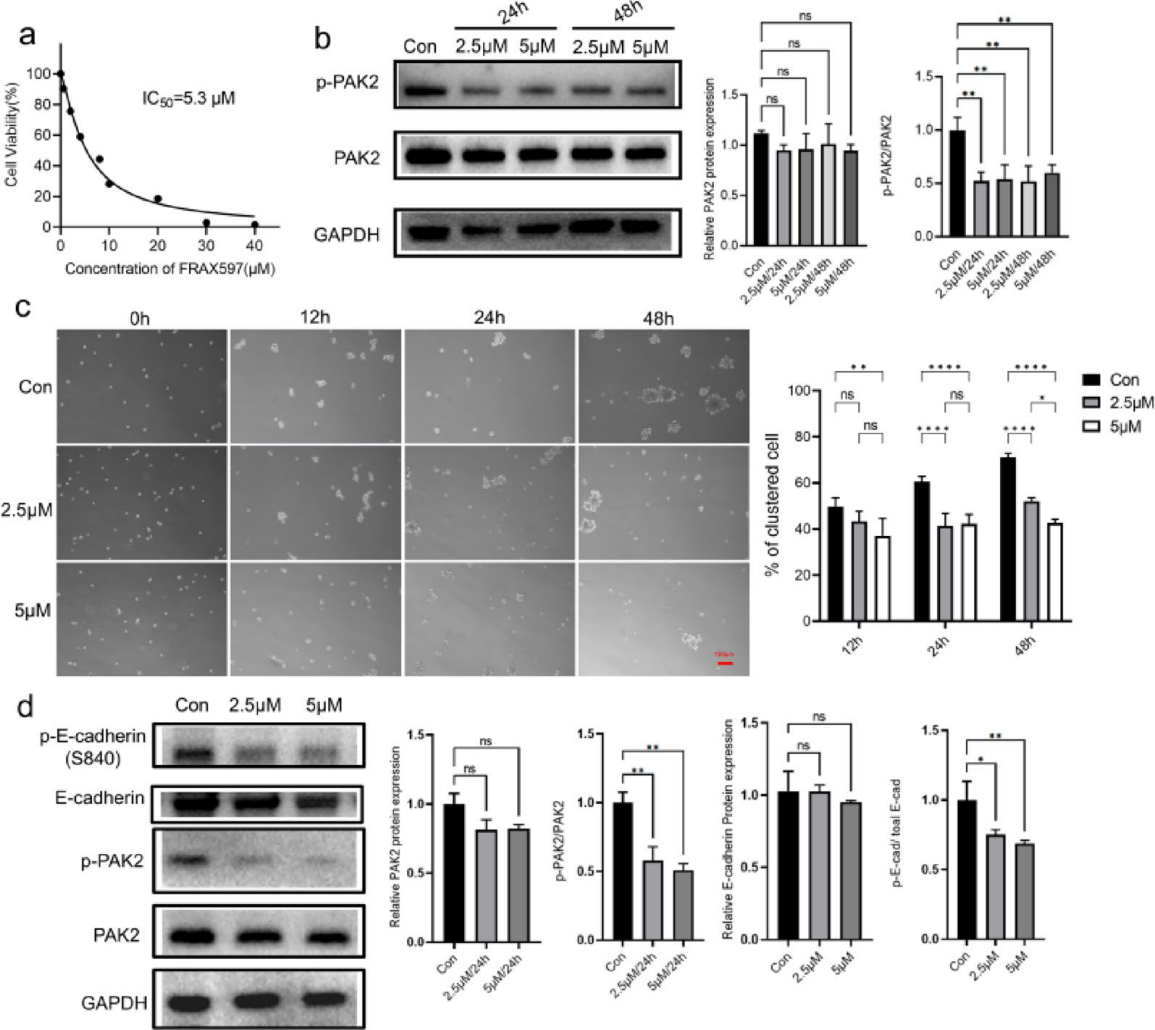
FRAX597 inhibits PAK2 activity and CTC cluster formation. **a** CCK-8 assay evaluating the viability of BT549 after 48 h treatment with FRAX597. **b** Immunoblot analysis of total PAK2 and phosphorylated PAK2 protein levels in BT549 treated with 2.5 or 5 µM FRAX597 for 24 and 48 h. **c** Representative images and quantification of BT549 cell aggregation after 24 h FRAX597 treatment. **d** Immunoblot analysis of total and phosphorylated PAK2, and total and phosphorylated E-cadherin in BT549 treated with 2.5 or 5 µM FRAX597 for 24 h

Our results above demonstrated that PAK2 induced the formation of cell clusters. Thus, we employed the potent PAK2 activity inhibitor FRAX597, a small-molecule pyridopyrimidinone, to determine the effect of PAK2 on cell aggregation. We detected the effect of FRAX597 on the viability of BT549 cells. The IC_50_ value of FRAX597 was 5.3 µM (Fig. 8a), and concentrations of 2.5 and 5 µM FRAX597 were used in subsequent experiments. Since phosphorylation of PAK2 at Ser141 reflects its kinase activity [31], we examined total PAK2 and p-PAK2 (Ser141) levels in BT549 cells treated with 2.5 or 5 µM FRAX597 for 24–48 h. The results showed that a 24-hour treatment was sufficient to markedly reduced PAK2 phosphorylation without affecting total PAK2 expression, and extending the treatment to 48 h did not result in further inhibition (Fig. 8b). Based on these findings, we selected 2.5 or 5 µM FRAX597 for 24 h as the optimal condition for subsequent functional assays. Under this condition, FRAX597 significantly suppressed CTC cluster formation (Fig. 8c), and immunoblotting further demonstrated that inhibition of PAK2 phosphorylation decreased E-cadherin phosphorylation (Fig. 8d). These findings demonstrate that FRAX597 reduces cell aggregation by inhibiting PAK2 activity and further confirm that PAK2 mediates cell cluster formation via phosphorylation of E-cadherin.

### Inhibition of PAK2 with FRAX597 significantly inhibits breast cancer metastasis in vitro and in vivo

**Fig. 9:**
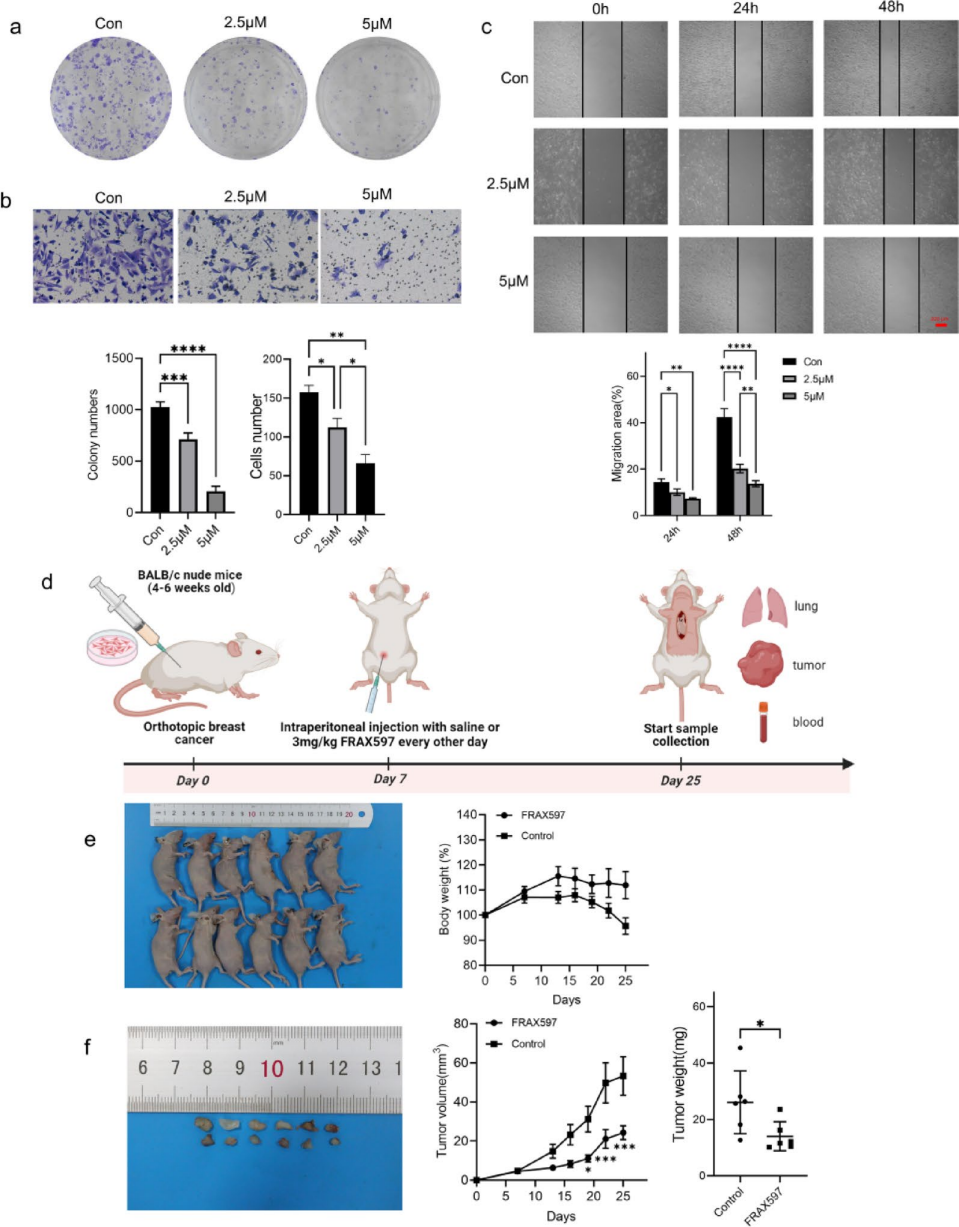
FRAX597 suppresses breast cancer progression. **a**–**c** Effects of FRAX597 on BT549 cell proliferation, migration and invasion. **d** Schematic diagram of in vivo experimental design. **e**, **f** Tumor volume, tumor weight and body weight measurements of nude mice treated with FRAX597

**Fig. 10:**
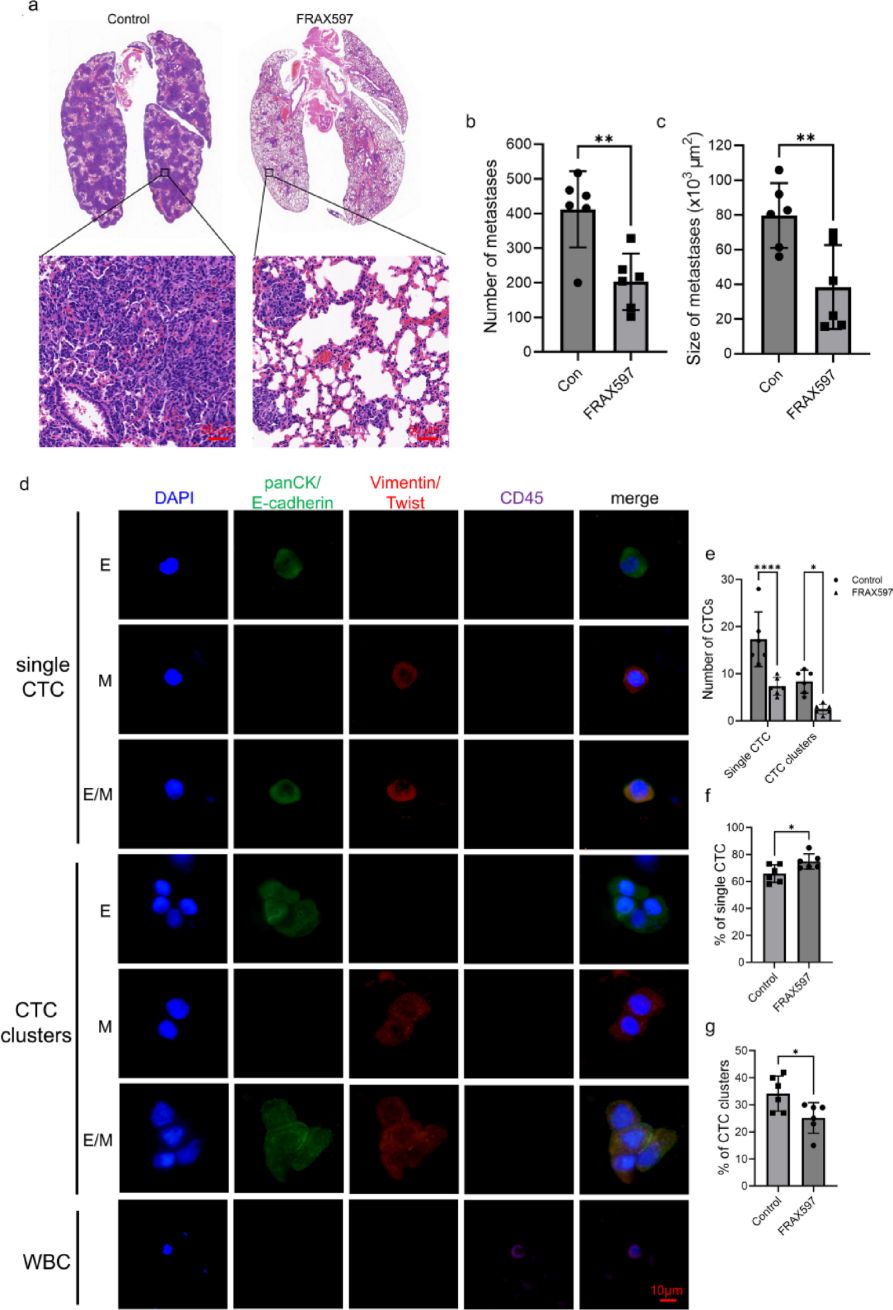
FRAX597 reduces breast cancer metastasis in vivo. **a** Representative H&E staining images of lung tissue sections. **a**–**c** Quantification of the number and size of metastatic nodules in the lungs. **d** Representative images of three phenotypes of CTCs detected in mouse blood. **e**–**g** Quantification of the number and percentage of single CTCs and CTC clusters in both groups

**Fig. 11:**
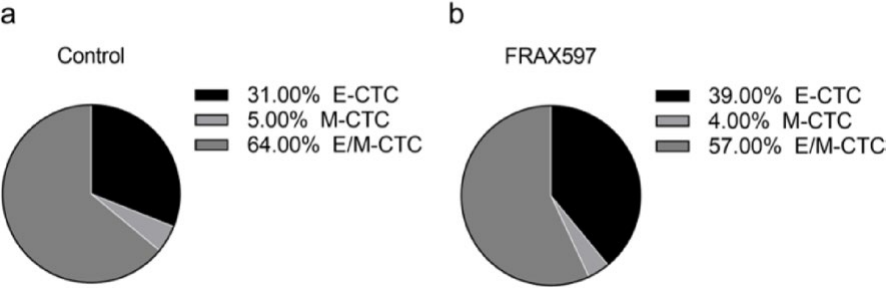
FRAX597 decreases the proportion of E/M-CTCs (**a**) Ratios of E-CTC, M-CTC and E/M-CTC in the control group. **b** Ratios of E-CTC, M-CTC and E/M-CTC in the FRAX597 treatment group

The inhibitor FRAX597 could significantly reduce the cluster formation in breast cancer cells, so we were interested in investigating its antimetastatic potential. Colony formation, wound healing, and Transwell assays demonstrated that FRAX597 treatment significantly suppressed BT549 cell proliferation, migration, and invasion (Fig. 9a, b and c). Next, in vivo experiments were conducted using female BALB/c nude mice, following the protocol outlined in Fig. 9d. Compared to the control group, tumor volume and weight were significantly reduced in the FRAX597-treated group, with no significant changes in mouse body weigh (Fig. 9e, f). And H&E staining revealed that the number and size of lung metastatic nodules were dramatically decreased in the FRAX597 treatment group (Fig. 10a, c). In addition, the number of CTCs in mouse blood was also detected by ISET to further investigate the ability of FRAX597 to reduce cell cluster formation. CTCs exhibit distinct phenotypes due to epithelial-mesenchymal transition (EMT), including epithelial CTCs (E-CTCs), mesenchymal CTCs (M-CTCs) and hybrid epithelial/mesenchymal CTCs (E/M-CTCs) [32]. To avoid potential bias, we performed multiplexed IF staining to quantify all CTC phenotypes (Fig. 10d). The results showed that FRAX597 treatment significantly reduced both single CTCs and CTC clusters in the blood of the mice (Fig. 10e). Notably, FRAX597 treatment further decreased the proportion of CTC clusters, suggesting that PAK2 inhibition can suppress tumor cell aggregation (Fig. 10f, g). Additionally, FRAX597 treatment also reduced the proportion of E/M-CTCs (Fig. 11a, b). Since partial EMT or a hybrid E/M phenotype is known to confer multiple advantages over a complete EMT phenotype, including facilitating CTC cluster formation [33], this finding suggests that FRAX597 may inhibit metastasis through multiple mechanisms. Collectively, our data demonstrate that PAK2 inhibition via FRAX597 effectively suppresses CTC cluster formation and metastatic potential, providing strong preclinical support for the targeting of PAK2 in breast cancer therapy.

**Fig. 12:**
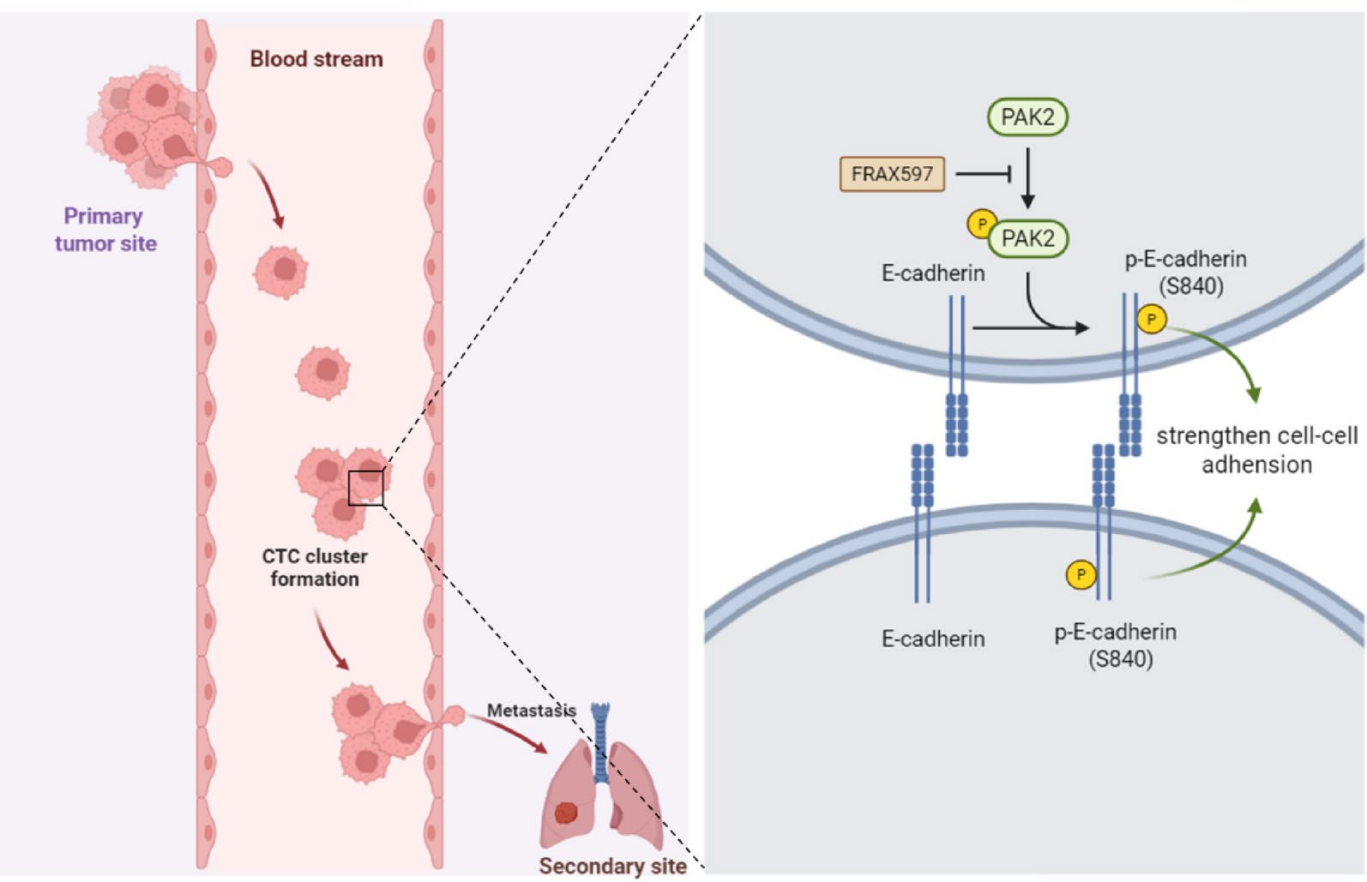
Schematic diagram. PAK2-mediated phosphorylation of E-cadherin at Ser840 promotes CTC clustering and metastasis

## Discussion

CTC clusters are recognized as highly metastatic intermediates in cancer dissemination and are strongly associated with poor clinical outcomes in patients with breast cancer. Despite their clinical relevance, the molecular mechanisms underlying CTC cluster formation remain incompletely understood. In this study, through integrative transcriptomic analyses of single CTCs and CTC clusters combined with molecular function studies, we explored for the first time the role of PAK2 in facilitating CTC cluster formation and metastatic progression in breast cancer. Our data demonstrate that PAK2-mediated phosphorylation of E-cadherin at Ser840 enhances cell-cell adhesion, and pharmacological inhibition of PAK2 by FRAX597 significantly impaired cluster formation and suppressed metastasis in vivo, indicating that PAK2 is a promising therapeutic target.

In our study, *PAK2* emerged as a key hub gene associated with CTC cluster formation based on our bioinformatics analysis. PAK2 is a serine/threonine kinase belonging to the p21-activated kinase family, and is known to regulate a variety of cellular functions, including cell survival, cytoskeletal remodeling, motility and proliferation [34]. Prior studies have shown that elevated PAK2 expression in multiple advanced cancers is closely associated with tumor progression and poor clinical outcomes [35]. In breast cancer specifically, PAK2 has been reported to modulate apoptosis through caspase-7 phosphorylation [31]. Beyond these functions, PAK2 promotes metastatic dissemination, angiogenesis, immune regulation and metabolic reprogramming through several oncogenic pathways, including Wnt/β-catenin, EGFR/HER2/MAPK and TGF-β signaling [36]. Consistent with these findings, we demonstrated that PAK2 overexpression enhanced proliferation migration and invasion of breast cancer cells, whereas PAK2 knockdown suppressed these metastatic phenotypes. Importantly, our study extends this knowledge by establishing a direct link between PAK2 and the formation of breast cancer CTC clusters.

Accumulating evidence also supports a role for PAK2 in cell adhesion. PAK2 contributes to the formation of new focal adhesions and regulates focal adhesion size [37]. In addition, PAK2 could form a signaling complex with Scrib and βPIX at adherens junctions, promote cell survival under physiological stress, and prevent anoikis [15]. Moreover, mechanical force applied to E-cadherin adhesion sites can recruit and activate PAK2, allow cells to maintain junctional stability, stiffen, metabolize and survive [16]. Together, these studies highlight a functional association between PAK2 and E-cadherin-mediated adhesion. E-cadherin plays a central role in adherens junctions, and its serine phosphorylation modulates cell-cell adhesion, thereby influencing tumor aggregation and metastasis [38]. Specifically, phosphorylation at Ser840 (likely mediated by CKII or GSK-3β) increases the binding affinity for β-catenin and strengthens the cell-cell adhesion [29, 30]. By integrating computational docking, IF colocalization, Co-IP assays and functional studies, our study revealed that PAK2 directly interacts with E-cadherin and phosphorylates it at Ser840, thereby strengthening intercellular adhesion and promoting CTC cluster formation in breast cancer. To our knowledge, this is the first demonstration that PAK2 modifies E-cadherin to promote tumor cell aggregation. These results support prior findings in epithelial cancers, where PAK kinases were shown to regulate junctional remodeling and mechanotransduction.

Among the candidate genes associated with CTC cluster formation, *JUP* and *ITGB3* were ranked after *PAK2* (Table S3). JUP (junction plakoglobin) is a core desmosomal component and adhesion molecule with established roles in CTC cluster formation and metastasis in breast cancer [3, 39]. ITGB3 (integrin β3) is a cell-surface adhesion receptor involved in tumor progression [40]. Although a direct link between ITGB3 and CTC clusters is unreported, its elevated expression correlates with poorer survival in breast cancer patients (Fig. S1-S2). Together, these findings suggest that both genes may contribute to adhesion-related processes relevant to CTC clusters and could potentially cooperate with PAK2, needing further investigation.

In KEGG analysis, enrichment of apoptosis, cell cycle and focal adhesion pathways, consistent with known characteristics of CTC clusters. Ubiquitin-mediated proteolysis and endocytosis pathways are also noteworthy because these processes has been reported to regulate the stability and localization of adhesion molecules [41, 42]. Dysregulation of these processes may influence the formation and plasticity of CTC clusters. Platelet activation pathway, supporting platelet-CTC interactions and protecting CTCs from shear stress and immune surveillance, may further stabilize clusters during circulation [43]. These findings suggest that CTC clusters may rely on coordinated regulatory mechanisms to maintain their integrity and metastatic potential.

Since CTC clusters are more aggressive than single CTCs, targeting them represents a promising therapeutic strategy for preventing metastasis. Choi et al. reported that treatment with urokinase effectively inhibited CTC cluster formation and prolonged overall survival by approximately 20% in mice [44]. A proof-of-concept phase Ⅰ trial demonstrated that inhibition of Na^+^/K^+^ ATPase could reduce CTC cluster size in metastatic breast cancer patients [45]. In preclinical studies, PAK2 inhibition also has shown potent antitumor activity, including suppression of breast cancer cell proliferation, reduced tumor growth in vivo, and inhibition of metastatic phenotypes [46, 47]. Consistently, our study revealed that FRAX597 significantly inhibited PAK2 phosphorylation and effectively reduced breast cancer cell clustering, invasion, migration, and metastatic spread both in vitro and in vivo. Notably, FRAX597 treatment reduced the number and proportion of CTC clusters, which are strongly associated with metastatic competence. Furthermore, in orthotopic breast cancer mouse models, systemic administration of FRAX597 not only suppressed primary tumor growth but also markedly diminished the metastatic burden in the lungs, reinforcing the potential clinical utility of PAK2 inhibition. Together, these findings suggest that PAK2 could serve as a novel target to limit metastasis in breast cancer, and pharmacological PAK2 inhibitors such as FRAX597 could provide multiple therapeutic benefits, including limiting tumor progression, reducing metastasis and improving patient outcomes. Despite these promising prospects, no PAK2 inhibitors have yet reached clinical use. One major challenge is that PAKs participate in numerous essential signaling pathways, and most currently available inhibitors target multiple PAK isoforms, raising concerns regarding off-target effects and systemic toxicity, such as cardiotoxicity [48]. Pharmacokinetics, membrane permeability and long-term safety of PAK2 inhibitors also remain insufficiently characterized. Development of isoform-selective inhibitors or improved delivery systems may facilitate clinical translation in breast cancer.

In addition, targeting PAK2 may also combine with standard treatments to enhance therapeutic efficacy. Aberrant activation of PAK2 has been strongly associated with therapeutic resistance in breast cancer, and its inhibition has been shown to suppress the development of multidrug resistance [49, 50]. Nevertheless, rational combination approaches will require further mechanistic studies and in vivo validation to ensure both efficacy and safety.

Despite the significance of our findings, several limitations should be noted. First, cell lines and mouse models cannot fully recapitulate the human tumor microenvironment. Validation using patient-derived xenografts and clinical samples will be necessary. Second, our work focuses on homotypic CTC clusters. The mechanisms driving heterotypic CTC cluster formation remain unexplored and warrant future investigation.

## Conclusion

In summary, our study identifies PAK2 as a critical regulator of CTC cluster formation and breast cancer metastasis. We demonstrate that PAK2 promotes CTC clustering through its kinase activity, which enhances E-cadherin phosphorylation at Ser840 and strengthens cell-cell adhesion, ultimately contributing to breast cancer metastasis (Fig. 12). The preclinical efficacy of the PAK inhibitor FRAX597 in disrupting this axis further supports the therapeutic potential of targeting PAK2 in metastatic breast cancer. Moving forward, validating these findings in clinical cohorts and elucidating the broader regulatory networks of PAK2 will be essential. Ultimately, therapeutic strategies aimed at reducing CTC cluster formation by targeting key mediators such as PAK2 hold substantial promise for improving outcomes in patients with breast cancer.

## Supplementary Information

Below is the link to the electronic supplementary material.

Supplementary Material 1.

Supplementary Material 2.

Supplementary Material 3.

## Data Availability

The data of GEO, CCLE and HPA used in this study were downloaded from public databases that are described in the main text. Experiments data will be made available on request.

## Abbreviations

BP: Biological process
CC: Cell component
coDEGs: Common differentially expressed genes
CTCs: Circulating tumor cells
DEGs: Differentially expressed genes
DMFS: Distant metastasis-free survival
E-CTC: Epithelial circulating tumor cell
E/M-CTC: Epithelial-mesenchymal circulating tumor cell
EMT: Epithelial-mesenchymal transition
GEO: Gene expression omnibus
GO: Gene ontology
GSEA: Gene set enrichment analysis
H&E: Hematoxylin and eosin
IHC: Immunohistochemistry
IF: Immunofluorescence
i.p.: Intraperitoneal injection
KEGG: Kyoto Encyclopedia of Genes and Genomes
M-CTC: Mesenchymal circulating tumor cell
MCC: Maximal clique centrality
MF: Molecular function
