# Supplementary material for: PAK2 promotes CTC cluster formation by phosphorylating E-cadherin to enhance cell-cell adhesion in breast cancer
Source: Breast Cancer Res. 2025 Dec 21;28:17. doi: 10.1186/s13058-025-02199-z (PMC12829041; doi:10.1186/s13058-025-02199-z)
Supplement: Supplementary file 1 — Supplementary Material 1. [file 13058_2025_2199_MOESM1_ESM.docx]

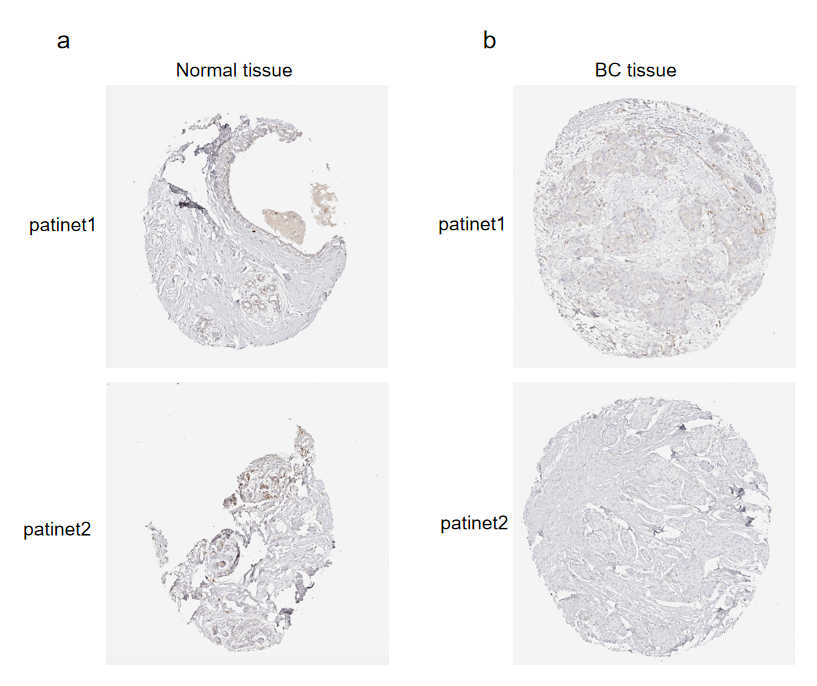


Figure S1. Representative IHC staining images of ITGB3 expression in normal breast tissues and BC tissues from the Human Protein Atlas database. (a) normal breast tissue; (b) BC tissues.


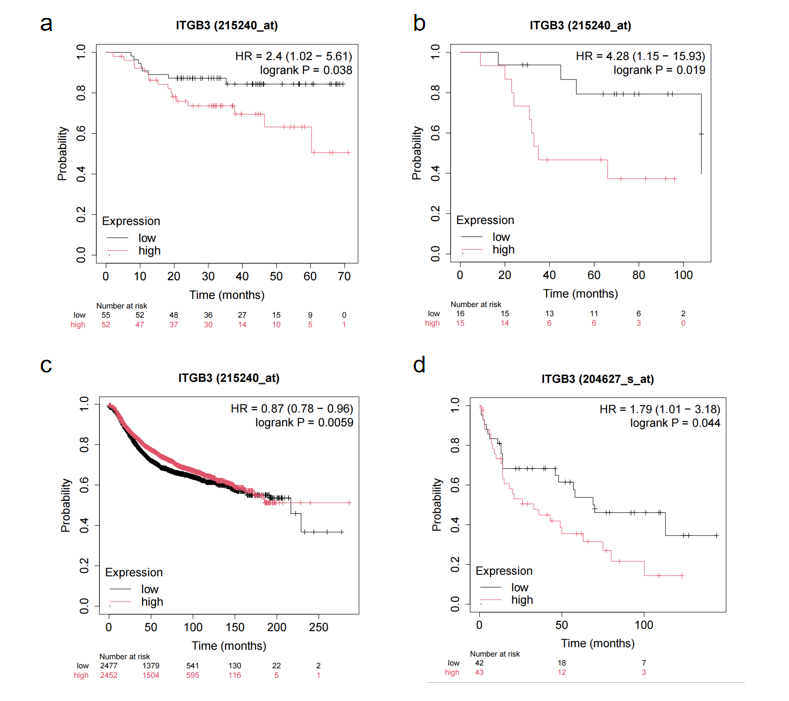


Figure S2. Kaplan-Meier analysis of the prognostic impact of ITGB3 expression in BC patients.(a) distant metastasis-free survival. (b)overall survival. (c)relapse-free survival. (d)post-progression survival.

Table S1. The clinical characteristics of breast patients in GSE51827 and GSE111065

| GEO datasets | Total number of patients | Total CTC number | Patient ID | Age | Stage | Histological type | Number of CTC samples per patient (RNA-seq) |
| --- | --- | --- | --- | --- | --- | --- | --- |
| GSE111065 | 12 | 69 (48 single CTCs and 21 CTC clusters) | Br37 | 53 | IV | Luminal (ER+/PR+) | two single CTCs |
|  |  |  | Br38 | 54 | IV | Luminal (ER+/PR+) | three single CTC |
|  |  |  | Br23 | 64 | IV | Luminal type(ER+) | one single CTC |
|  |  |  | Br44 | 51 | IV | HER2+ | one single CTC/four CTC clusters |
|  |  |  | Br39 | 53 | IV | Luminal (ER+/PR+) | four single CTC/one CTC clusters |
|  |  |  | Br11 | 58 | IV | triple-negative | eleven single CTCs/two CTC clusters |
|  |  |  | Br45 | 51 | IV | HER2+ | two single CTCs |
|  |  |  | Br57 | 56 | IV | Luminal (ER+) | thirteen single CTCs/one CTC clusters |
|  |  |  | Br61 | 63 | IV | Luminal (ER+) | seven single CTCs/seven CTC clusters |
|  |  |  | Br7 | 42 | IV | Luminal (ER+/PR+) | two single CTCs/two CTC clusters |
|  |  |  | Br16 | 49 | IV | Luminal (ER+/PR+) | one single CTC/four CTC clusters |
|  |  |  | Br53 | 59 | IV | Luminal (ER+) | one single CTC/four CTC clusters |
| GSE51827 | 10 | 29 (15 single CTCs and 14 CTC-clusters) | Brx53 | NA | IV | Luminal type: n = 6;  triple-negative: n = 2;  HER2+: n = 2 | one single CTC/one CTC clusters |
|  |  |  | BrTr08 | NA | IV |  | one single CTC/one CTC clusters |
|  |  |  | Brx12 | NA | IV |  | one single CTC/one CTC clusters |
|  |  |  | Brx52 | NA | IV |  | two single CTCs/three CTC clusters |
|  |  |  | Brx17 | NA | IV |  | two single CTCs/one CTC clusters |
|  |  |  | Brx39 | NA | IV |  | one single CTC/one CTC clusters |
|  |  |  | Brx66 | NA | IV |  | two single CTCs/one CTC clusters |
|  |  |  | Brx61 | NA | IV |  | three single CTCs/two CTC clusters |
|  |  |  | Brx11 | NA | IV |  | one single CTC/one CTC clusters |
|  |  |  | BrTr11 | NA | IV |  | one single CTC/two CTC clusters |

Table S2. The detail information of coDEGs between CTC clusters an single CTCs

| GENE | baseMean | log2FoldChange | lfcSE | stat | padj | Change |
| --- | --- | --- | --- | --- | --- | --- |
| *DUSP22* | 60.32651011 | -2.099554004 | 0.953148159 | -2.202757236 | 0.027611864 | down |
| *TNFAIP8* | 88.23853171 | -2.125553275 | 0.924247373 | -2.299766639 | 0.021461444 | down |
| *TMEM91* | 10.56950637 | -2.285205474 | 1.063128873 | -2.149509371 | 0.031594044 | down |
| *TUBA4A* | 270.3735955 | -2.305234876 | 0.880342983 | -2.618564492 | 0.00883006 | down |
| *RNF145* | 48.21835914 | -2.461445467 | 0.770422681 | -3.194928613 | 0.001398654 | down |
| *MXI1* | 29.69708568 | -2.470293775 | 0.981153737 | -2.517743838 | 0.011810918 | down |
| *NFATC1* | 34.51894966 | -2.827583977 | 1.275204016 | -2.217358118 | 0.026598629 | down |
| *SCD* | 123.0347342 | -2.864581003 | 1.251872694 | -2.288236669 | 0.022123742 | down |
| *BIRC3* | 59.90509737 | -2.922582229 | 1.09064206 | -2.67968964 | 0.007369045 | down |
| *PPBP* | 39.99000269 | -3.000600023 | 1.284277726 | -2.336410546 | 0.019469855 | down |
| *GAB2* | 358.5388563 | -3.087978909 | 1.268848916 | -2.433685264 | 0.014945985 | down |
| *CALHM2* | 24.31927746 | -3.518904092 | 1.471520417 | -2.391338952 | 0.016787047 | down |
| *PHLDA1* | 142.8079536 | -3.704484476 | 1.413125099 | -2.621483745 | 0.008754794 | down |
| *PGM2L1* | 704.2773935 | -3.854517538 | 0.908396555 | -4.243210211 | 2.20E-05 | down |
| *CCNJL* | 43.98160866 | -3.979660128 | 1.242478598 | -3.203000947 | 0.001360035 | down |
| *CYTH3* | 28.1549298 | -4.000537974 | 1.121347398 | -3.567616941 | 0.000360243 | down |
| *OVGP1* | 10.81061273 | -4.020304194 | 1.713657326 | -2.346037409 | 0.018974203 | down |
| *RGS18* | 23.38028361 | -4.038017578 | 1.529594986 | -2.639926004 | 0.008292413 | down |
| *VSIG2* | 26.24870989 | -4.101379519 | 1.645374139 | -2.492672896 | 0.012678559 | down |
| *PF4* | 29.6436056 | -4.387143035 | 1.115849234 | -3.9316629 | 8.44E-05 | down |
| *SH3BGRL2* | 29.41110598 | -4.442326309 | 1.253008578 | -3.54532793 | 0.000392125 | down |
| *KIF3C* | 68.284245 | -4.607818647 | 1.387336205 | -3.321342462 | 0.000895855 | down |
| *SLC16A1* | 9.929104479 | -4.624029105 | 1.814732827 | -2.548049519 | 0.010832709 | down |
| *ROBO1* | 70.3067228 | -4.79431101 | 1.271884486 | -3.769454744 | 0.000163605 | down |
| *PCP2* | 7.492006134 | -4.869481823 | 1.616430854 | -3.012490025 | 0.00259114 | down |
| *F13A1* | 31.35713006 | -5.05646187 | 2.41442815 | -2.094268935 | 0.03623603 | down |
| *KPNA5* | 434.0176998 | -5.339296471 | 1.192580536 | -4.477095099 | 7.57E-06 | down |
| *ACRBP* | 6.488784657 | -5.371143307 | 2.162467842 | -2.483802627 | 0.012998783 | down |
| *GP9* | 6.853002503 | -5.436813575 | 2.631462974 | -2.06608021 | 0.038820907 | down |
| *ICAM2* | 11.19295599 | -5.741407809 | 2.393547914 | -2.39870185 | 0.016453305 | down |
| *SDPR* | 32.84672922 | -6.35439888 | 1.679158952 | -3.784274784 | 0.000154157 | down |
| *CD69* | 14.27571551 | -6.503412779 | 3.165357779 | -2.054558516 | 0.039921666 | down |
| *PLA2G2A* | 52.51903123 | -7.188964326 | 2.302715048 | -3.121951338 | 0.001796566 | down |
| *GPR183* | 9.517740372 | -8.849732698 | 3.167598562 | -2.793830255 | 0.005208781 | down |
| *GALNT14* | 14.31134968 | -8.990904676 | 2.213837735 | -4.061230203 | 4.88E-05 | down |
| *CYP3A5* | 264.6422697 | -10.82838022 | 1.523410955 | -7.107983686 | 1.18E-12 | down |
| *DAPL1* | 7.23095531 | -22.30381904 | 2.749627826 | -8.11157744 | 5.00E-16 | down |
| *ITGB3* | 14.26773031 | 7.463226221 | 6.785891657 | -1.21902506 | 1.89E-07 | up |
| *ITGA2B* | 20.1231833 | 7.363507152 | 6.910144846 | -0.625068436 | 1.88E-09 | up |
| *GRB14* | 7.223602173 | 6.888802235 | 1.803779785 | 3.819092715 | 0.000133943 | up |
| *DAB2* | 22.39163537 | 5.816000183 | 1.305150226 | 4.456192144 | 8.34E-06 | up |
| *ZFP2* | 5.127806404 | 5.418482133 | 2.279782569 | 2.376753909 | 0.017465736 | up |
| *TOP2A* | 51.1857472 | 5.413689102 | 1.809264338 | 2.992204615 | 0.002769706 | up |
| *PRIM1* | 31.68699599 | 4.645679309 | 1.286364299 | 3.611480288 | 0.000304454 | up |
| *ZNF662* | 6.798068494 | 4.354497436 | 1.727384907 | 2.520861111 | 0.011706805 | up |
| *SLMO1* | 18.10446864 | 4.11286947 | 1.490590457 | 2.759221657 | 0.005793922 | up |
| *SLX4* | 15.74875082 | 4.021661435 | 1.538576932 | 2.613883876 | 0.008951946 | up |
| *CCNB1* | 28.2656464 | 3.914834594 | 1.652489351 | 2.369052842 | 0.017833705 | up |
| *RCN1* | 150.5772946 | 3.734477084 | 0.923716265 | 4.042883324 | 5.28E-05 | up |
| *SLC16A5* | 35.16088317 | 3.695789186 | 1.387511724 | 2.66360934 | 0.007730728 | up |
| *ACER3* | 106.510985 | 3.663541889 | 0.90874114 | 4.031447159 | 5.54E-05 | up |
| *ZNF346* | 184.5897499 | 3.63261857 | 0.971070091 | 3.740840752 | 0.000183406 | up |
| *CMAHP* | 256.8232416 | 3.629936992 | 1.060598937 | 3.422535009 | 0.000620401 | up |
| *MB21D2* | 99.88908636 | 3.615931212 | 1.397454135 | 2.587513337 | 0.009667145 | up |
| *FAM3B* | 112.0039779 | 3.612174314 | 1.140486222 | 3.167223105 | 0.001539022 | up |
| *PPP1R14C* | 136.9913401 | 3.527627504 | 1.095072266 | 3.22136503 | 0.001275815 | up |
| *C15orf38* | 109.8430018 | 3.517780659 | 0.797700927 | 4.409899173 | 1.03E-05 | up |
| *PPP1R1B* | 128.8663021 | 3.507873859 | 1.359997343 | 2.579324054 | 0.009899387 | up |
| *SCGB1D2* | 191.8155987 | 3.491287628 | 1.752952143 | 1.99166169 | 0.04640819 | up |
| *SLC45A4* | 82.86346683 | 3.481052447 | 1.010263115 | 3.445688946 | 0.000569606 | up |
| *PHACTR2* | 90.28402964 | 3.387532582 | 0.974368176 | 3.476645342 | 0.000507729 | up |
| *LGALS3BP* | 1177.37258 | 3.261412119 | 0.831568355 | 3.92200124 | 8.78E-05 | up |
| *FZD1* | 24.82436785 | 3.257040636 | 1.265525974 | 2.573665576 | 0.010062749 | up |
| *MAML1* | 152.8266437 | 3.238816732 | 0.848400493 | 3.817556398 | 0.00013478 | up |
| *IFI27* | 118.4988149 | 3.226094869 | 1.238029115 | 2.60583118 | 0.009165162 | up |
| *TCEANC2* | 13.6517641 | 3.19879923 | 1.595416932 | 2.004992655 | 0.044963832 | up |
| *GRK6* | 158.1545534 | 3.18711461 | 0.746715831 | 4.268176027 | 1.97E-05 | up |
| *KRCC1* | 65.95953888 | 3.175999634 | 0.894715994 | 3.549729361 | 0.000385627 | up |
| *SLC12A7* | 431.9511885 | 3.168650558 | 0.80820812 | 3.920587382 | 8.83E-05 | up |
| *DEAF1* | 55.53556241 | 3.151702169 | 0.82270678 | 3.830893639 | 0.000127679 | up |
| *FERMT3* | 28.36866671 | 3.127406731 | 5.533122185 | 3.544776949 | 0.000194379 | up |
| *CITED4* | 78.62701601 | 3.081283362 | 1.092280874 | 2.820962478 | 0.00478798 | up |
| *ZNF689* | 21.46976531 | 3.064980189 | 1.535217848 | 1.996446429 | 0.045885351 | up |
| *STEAP4* | 226.5283127 | 3.063671078 | 1.300628839 | 2.355530637 | 0.018496272 | up |
| *LY6E* | 623.4861387 | 3.036811869 | 0.90346319 | 3.361301162 | 0.000775762 | up |
| *KIFC2* | 75.93048943 | 3.033706938 | 0.803863665 | 3.773907282 | 0.000160711 | up |
| *C4orf19* | 82.04179619 | 2.994915695 | 1.033227964 | 2.898601083 | 0.003748315 | up |
| *PYCR1* | 659.2100809 | 2.993586989 | 0.709861361 | 4.217143171 | 2.47E-05 | up |
| *CA5B* | 44.83775705 | 2.993353396 | 0.918885075 | 3.257592793 | 0.001123615 | up |
| *SEPT11* | 176.9720787 | 2.986283229 | 0.803812366 | 3.715149648 | 0.000203083 | up |
| *SEC22A* | 55.74985802 | 2.980902308 | 0.969705368 | 3.074028881 | 0.00211189 | up |
| *HNRNPAB* | 230.533391 | 2.931152706 | 0.572247471 | 5.12217678 | 3.02E-07 | up |
| *CNTNAP2* | 35.90966838 | 2.922063592 | 1.333020873 | 2.192061393 | 0.028375074 | up |
| *C9orf85* | 45.95469944 | 2.909820493 | 0.927283264 | 3.13800605 | 0.001701014 | up |
| *PLA2G16* | 186.1416135 | 2.897749935 | 0.744032143 | 3.894656919 | 9.83E-05 | up |
| *ZNF117* | 60.18916306 | 2.876093311 | 0.927171179 | 3.102008968 | 0.001922121 | up |
| *ZNF490* | 19.52438987 | 2.862366244 | 1.295316163 | 2.209781925 | 0.027120301 | up |
| *ZBTB1* | 128.7371714 | 2.855599953 | 0.781847319 | 3.65237545 | 0.000259826 | up |
| *PTPRE* | 37.3915929 | 2.838300777 | 1.253706836 | 2.263927016 | 0.023578604 | up |
| *EMC2* | 122.5759476 | 2.835423612 | 0.852687382 | 3.325279197 | 0.0008833 | up |
| *L2HGDH* | 40.35722641 | 2.812697995 | 1.136109644 | 2.475727594 | 0.013296498 | up |
| *ZNF720* | 21.38149313 | 2.812625235 | 1.054680304 | 2.666803604 | 0.007657641 | up |
| *SEZ6L2* | 27.52672939 | 2.738168483 | 1.080426214 | 2.53434103 | 0.011265903 | up |
| *CKAP5* | 112.0657998 | 2.729396657 | 0.736397799 | 3.706416097 | 0.000210213 | up |
| *CTSF* | 177.0202223 | 2.72466118 | 0.889534596 | 3.063018788 | 0.002191163 | up |
| *MNS1* | 10.4945743 | 2.708840399 | 1.250945921 | 2.165433657 | 0.030354491 | up |
| *KIN* | 46.45442728 | 2.70502512 | 0.918729159 | 2.944311818 | 0.003236737 | up |
| *DTNB* | 71.09777364 | 2.703180801 | 0.882455006 | 3.063250572 | 0.002189466 | up |
| *MRPL46* | 69.2106627 | 2.694916629 | 0.873616669 | 3.084781604 | 0.002037017 | up |
| *ALDH2* | 275.0954026 | 2.677441904 | 0.898958334 | 2.978382648 | 0.00289774 | up |
| *MAP3K5* | 127.2185745 | 2.676682309 | 0.92685102 | 2.887931556 | 0.003877842 | up |
| *EIF1* | 4471.325095 | 2.666720774 | 0.560413838 | 4.758484878 | 1.95E-06 | up |
| *SLC39A14* | 47.96276476 | 2.66092923 | 1.045764319 | 2.544482712 | 0.010943973 | up |
| *KIAA1244* | 517.1466331 | 2.643707511 | 0.673349256 | 3.926205437 | 8.63E-05 | up |
| *CPNE2* | 23.73228677 | 2.629691181 | 1.318666286 | 1.994205212 | 0.046129633 | up |
| *RUFY1* | 201.6115949 | 2.623748417 | 0.720152727 | 3.643322201 | 0.000269142 | up |
| *ANKRD13A* | 67.22939247 | 2.590956162 | 0.724329087 | 3.577042822 | 0.000347503 | up |
| *CTNNBL1* | 115.5311601 | 2.586348683 | 0.798261206 | 3.239977921 | 0.00119539 | up |
| *DSN1* | 89.9575866 | 2.58297212 | 1.031202148 | 2.504816467 | 0.012251495 | up |
| *POGLUT1* | 68.26806163 | 2.556514931 | 1.147753515 | 2.227407625 | 0.025920043 | up |
| *PPIL4* | 49.43179023 | 2.542859542 | 0.904430309 | 2.811559405 | 0.004930199 | up |
| *RNASEH2B* | 29.22874853 | 2.540211447 | 1.199251916 | 2.118163343 | 0.034161236 | up |
| *FZR1* | 155.35881 | 2.515015394 | 0.670149286 | 3.752918112 | 0.000174788 | up |
| *SORD* | 33.80273245 | 2.500937941 | 0.972052779 | 2.572841718 | 0.010086733 | up |
| *YEATS2* | 62.7282027 | 2.484412479 | 0.815177721 | 3.047694282 | 0.002306044 | up |
| *JUP* | 3449.054117 | 2.483961289 | 0.694975456 | 3.57417124 | 0.000351339 | up |
| *CTSH* | 241.2390204 | 2.474673549 | 0.759317554 | 3.259075909 | 0.001117758 | up |
| *IRS2* | 79.95354016 | 2.474617411 | 1.026460151 | 2.410826576 | 0.015916415 | up |
| *ST6GAL1* | 212.0414142 | 2.444438788 | 0.881464006 | 2.773157804 | 0.005551519 | up |
| *TACC1* | 118.5639168 | 2.443793939 | 0.908623088 | 2.689557388 | 0.007154684 | up |
| *PTPLAD1* | 648.9574255 | 2.439027778 | 0.65993324 | 3.695870475 | 0.000219135 | up |
| *KIF22* | 222.7655322 | 2.423514137 | 0.7635224 | 3.174123164 | 0.001502899 | up |
| *IFT122* | 70.46129182 | 2.41791297 | 0.922685503 | 2.620516917 | 0.008779657 | up |
| *SEPHS2* | 576.5758726 | 2.414553592 | 0.681559415 | 3.542689809 | 0.000396068 | up |
| *CLSTN1* | 312.1001479 | 2.412148747 | 0.661856882 | 3.644517136 | 0.000267894 | up |
| *SNHG8* | 137.0765955 | 2.41179627 | 0.697630009 | 3.457128045 | 0.000545965 | up |
| *SF3B5* | 289.1095031 | 2.409490184 | 0.645621116 | 3.732049843 | 0.000189928 | up |
| *DNAAF2* | 72.8829229 | 2.382400744 | 0.962106043 | 2.476235091 | 0.013277612 | up |
| *LCN2* | 138.4347006 | 2.359760991 | 1.137186606 | 2.075086867 | 0.037978485 | up |
| *CAPG* | 192.2592854 | 2.35622186 | 0.9937111 | 2.371133683 | 0.017733616 | up |
| *BLZF1* | 102.4071472 | 2.336426814 | 0.882047537 | 2.648867229 | 0.008076205 | up |
| *BST2* | 302.7449616 | 2.33604034 | 0.904918064 | 2.581493766 | 0.009837377 | up |
| *CBL* | 85.5669683 | 2.335327976 | 0.86738855 | 2.692366615 | 0.007094691 | up |
| *SIX4* | 29.78387613 | 2.332783756 | 1.081840997 | 2.156309257 | 0.031059525 | up |
| *NR2C1* | 53.83935049 | 2.31304462 | 0.922382763 | 2.507684135 | 0.012152525 | up |
| *FBXW8* | 27.40825882 | 2.306345724 | 1.025647674 | 2.248672505 | 0.02453334 | up |
| *NPAT* | 72.65285292 | 2.294160964 | 1.094350769 | 2.096367114 | 0.036049637 | up |
| *GALNT6* | 498.4842068 | 2.28913989 | 0.737426583 | 3.104227515 | 0.001907766 | up |
| *IVD* | 214.6372558 | 2.271762398 | 0.872884137 | 2.60259329 | 0.009252164 | up |
| *UBE2V2* | 121.355605 | 2.257916454 | 0.639700451 | 3.529646496 | 0.000416115 | up |
| *ERCC6L2* | 69.43213991 | 2.253723491 | 0.84271809 | 2.674350434 | 0.007487417 | up |
| *SH3KBP1* | 22.157919 | 2.248051484 | 0.718573783 | 3.12849082 | 0.001757065 | up |
| *PRKDC* | 334.8822362 | 2.242636469 | 0.696313659 | 3.220727382 | 0.001278657 | up |
| *FAM206A* | 94.7591895 | 2.242545599 | 0.822898445 | 2.725179045 | 0.006426663 | up |
| *ANKRD37* | 37.62947111 | 2.239053873 | 0.941292182 | 2.378702296 | 0.017373701 | up |
| *E2F3* | 72.68530848 | 2.235429398 | 0.967363019 | 2.310848517 | 0.020841224 | up |
| *MGLL* | 385.2287188 | 2.227080203 | 0.784163608 | 2.840070849 | 0.004510352 | up |
| *PAK2* | 158.4725184 | 2.223342333 | 0.544950366 | 4.079898783 | 4.51E-05 | up |
| *NPC2* | 423.051763 | 2.213654563 | 0.730928668 | 3.028550748 | 0.002457298 | up |
| *PTGFRN* | 154.8648372 | 2.206245244 | 0.794183656 | 2.778003837 | 0.005469397 | up |
| *ZNF252P* | 42.05894004 | 2.192390707 | 1.035561033 | 2.117104292 | 0.034250998 | up |
| *STUB1* | 198.0382986 | 2.191555167 | 0.607611032 | 3.606839001 | 0.00030995 | up |
| *AGPAT1* | 83.35555732 | 2.185553093 | 0.814885694 | 2.682036402 | 0.00731755 | up |
| *FAM109A* | 35.7661451 | 2.18315037 | 1.004856707 | 2.172598695 | 0.029810534 | up |
| *BET1* | 53.14423404 | 2.180531032 | 0.9375587 | 2.325754143 | 0.02003167 | up |
| *DNPEP* | 254.6258995 | 2.165752581 | 0.767591773 | 2.821490089 | 0.004780111 | up |
| *C9orf16* | 109.0610001 | 2.158518231 | 0.627018788 | 3.442509654 | 0.000576343 | up |
| *APLP2* | 777.2497093 | 2.157670659 | 0.657389134 | 3.282181814 | 0.001030072 | up |
| *PDZD2* | 97.994994 | 2.157554036 | 0.974459068 | 2.214104323 | 0.026821603 | up |
| *RNF6* | 102.7824683 | 2.153614154 | 0.768445531 | 2.80255928 | 0.00506989 | up |
| *C2orf49* | 39.34581049 | 2.149485085 | 0.867551746 | 2.477644815 | 0.013225273 | up |
| *PRPF4* | 129.8988269 | 2.142742631 | 0.834603618 | 2.567377598 | 0.010247096 | up |
| *SNX27* | 132.8898024 | 2.141639087 | 0.659527901 | 3.247230456 | 0.00116534 | up |
| *SPIRE2* | 25.41245432 | 2.139257738 | 1.046982982 | 2.043259322 | 0.041026774 | up |
| *FCHO2* | 88.23184939 | 2.122538274 | 0.730077266 | 2.90727896 | 0.003645879 | up |
| *PLK1S1* | 31.9148344 | 2.12027555 | 1.065867368 | 1.989248957 | 0.046673731 | up |
| *TMEM218* | 54.71312622 | 2.119825216 | 0.990220006 | 2.140761855 | 0.032293247 | up |
| *GMPPA* | 91.77806748 | 2.112227321 | 0.753792621 | 2.802133191 | 0.005076591 | up |
| *PIAS1* | 221.4559375 | 2.100107528 | 0.645816381 | 3.251864757 | 0.001146506 | up |
| *ZNF100* | 30.24259691 | 2.096837908 | 0.927433905 | 2.260902795 | 0.023765277 | up |
| *CHMP6* | 145.3075252 | 2.095778878 | 0.790232782 | 2.652103186 | 0.007999209 | up |
| *TIMMDC1* | 273.2627034 | 2.091916094 | 0.721452677 | 2.89958879 | 0.003736525 | up |
| *RGS19* | 46.99228855 | 2.06760082 | 0.932680845 | 2.216836371 | 0.026634275 | up |
| *CD81* | 142.6516326 | 2.065510668 | 0.641087741 | 3.221884517 | 0.001273505 | up |
| *RAB31* | 89.69108668 | 2.057462689 | 1.006119847 | 2.04494792 | 0.040859993 | up |
| *PERP* | 1399.998224 | 2.055297405 | 0.627421937 | 3.275781866 | 0.001053699 | up |
| *ANAPC7* | 182.4031884 | 2.049269809 | 0.681372858 | 3.00756008 | 0.002633541 | up |
| *SYCP2* | 175.3664202 | 2.048132816 | 0.893416413 | 2.292472788 | 0.021878376 | up |
| *HTATIP2* | 124.059731 | 2.043170073 | 0.873266669 | 2.339686313 | 0.019299942 | up |
| *NAA15* | 274.7652517 | 2.035791705 | 0.712782649 | 2.856118492 | 0.004288551 | up |
| *TOR1AIP2* | 461.4304612 | 2.03326614 | 0.567238166 | 3.584501648 | 0.000337722 | up |
| *C4orf27* | 30.00046133 | 2.033229572 | 0.989965302 | 2.053839229 | 0.039991255 | up |
| *EXPH5* | 100.403384 | 2.031594645 | 0.767126136 | 2.6483189 | 0.008089318 | up |
| *BICD2* | 108.4025194 | 2.028434136 | 0.860965785 | 2.355998545 | 0.01847299 | up |
| *SRF* | 53.51337276 | 2.016694847 | 0.860510967 | 2.343601562 | 0.019098562 | up |
| *PRRG4* | 133.7670457 | 2.014908419 | 0.776068736 | 2.596301494 | 0.009423334 | up |
| *RNF139* | 122.9426265 | 2.013734317 | 0.833520834 | 2.415937594 | 0.015694751 | up |
| *ZC3H15* | 361.9313787 | 2.009893684 | 0.600948061 | 3.344538096 | 0.000824197 | up |
| *FOXJ2* | 64.10612722 | 2.009511368 | 0.933541192 | 2.152568505 | 0.031352606 | up |
| *AP3S2* | 179.0851807 | 2.002788859 | 0.761602574 | 2.629703375 | 0.00854594 | up |
| *ZHX2* | 95.31716299 | 2.000924219 | 0.850446622 | 2.352792246 | 0.018633041 | up |

Table S3. Cell adhesion–related genes in Venn diagram intersections

| Item | Number | Gene |
| --- | --- | --- |
| GOTERM_BP_DIRECT_cell adhesion  GOTERM_CC_DIRECT_cell-cell junction  GOTERM_MF_DIRECTF_cadherin binding  MCC_SCORE | 1 | *PAK2* |
| GOTERM_BP_DIRECT_cell adhesion  GOTERM_CC_DIRECT_cell-cell junction  GOTERM_MF_DIRECTF_cadherin binding | 1 | *JUP* |
| GOTERM_BP_DIRECT_cell adhesion GOTERM_CC_DIRECT_cell-cell junction  MCC_SCORE | 1 | *ITGB3* |
| GOTERM_BP_DIRECT_cell adhesion GOTERM_CC_DIRECT_cell-cell junction | 1 | *PDZD2* |
| GOTERM_BP_DIRECT_cell adhesion  MCC_SCORE | 3 | *FERMT3, GP9, ITGA2B* |
| GOTERM_CC_DIRECTT_cell-cell junction  MCC_SCORE | 1 | *SH3KBP1* |
| GOTERM_MF_DIRECT_cadherin binding  MCC_SCORE | 2 | *CKAP5, CBL* |
| GOTERM_BP_cell adhesion | 5 | *ICAM2, CNTNAP2, ROBO1, CLSTN1, LGALS3BP* |
| GOTERM_CC_cell-cell junction | 1 | *PERP* |
| GOTERM_MF_cadherin binding | 3 | *MB21D2, CAPG, ZC3H15* |
| MCC_SCORE | 22 | *PHACTR2, CCNB1, TOP2A, SF3B5, KIF22, GPR183, DAB2, STUB1, CD81, F13A1, HTATIP2, PRKDC, PPBP, PF4, BIRC3, APLP2, LY6E, IFI27, FZR1,BST2, PRIM1 KIF3C* |
