# Supplementary material for: PAK2 promotes CTC cluster formation by phosphorylating E-cadherin to enhance cell-cell adhesion in breast cancer
Source: Breast Cancer Res. 2025 Dec 21;28:17. doi: 10.1186/s13058-025-02199-z (PMC12829041; doi:10.1186/s13058-025-02199-z)
Supplement: Supplementary file 2 — Supplementary Material 2. [file 13058_2025_2199_MOESM2_ESM.docx]

Figure 3g

70

55

40

35

25

100

PAK2 58 kDa

p-PAK2 58kDa

GAPDH 36 kDa

BT549

MDA-MB-231

AU565

SKBR3

Repeat 1

55

100

70


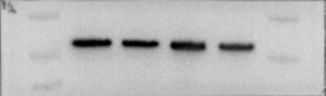

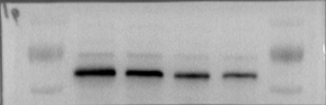

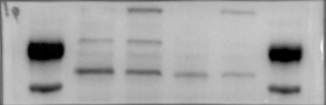


70

55

40

35

25

100

PAK2 58 kDa

p-PAK2 58 kDa

GAPDH 36 kDa

BT549

MDA-MB-231

AU565

SKBR3

Repeat 2

100

55

70


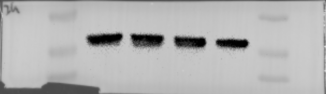

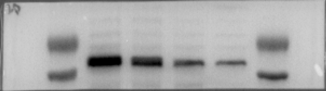

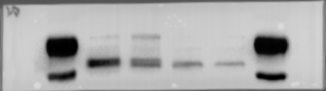

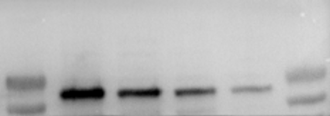

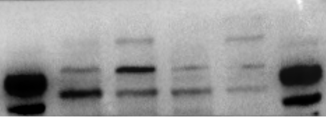

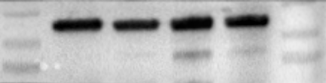


70

55

40

35

25

100

PAK2 58 kDa

p-PAK2 58kDa

GAPDH 36 kDa

BT549

MDA-MB-231

AU565

SKBR3

Repeat 3

100

55

70

Figure 4a

40

35

25

180

PAK2 58 kDa

GAPDH 36 kDa

Repeat 2

130

100

55

70

Con

Vector

OE


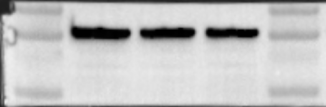

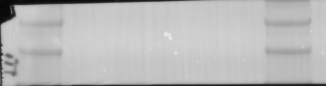

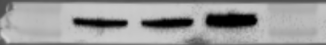

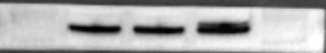


40

35

25

180

PAK2 58 kDa

GAPDH 36 kDa

Repeat 1

130

100

55

70


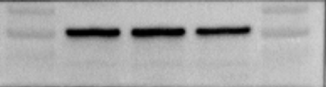

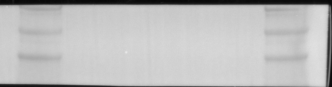


Con

Vector

OE

40

35

25

180

PAK2 58 kDa

GAPDH 36 kDa

Repeat 3

130

100

55

70

Con

Vector

OE


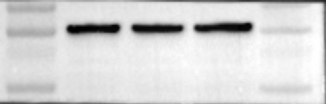

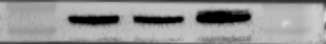

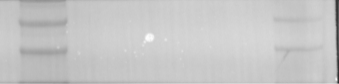


Figure 4b


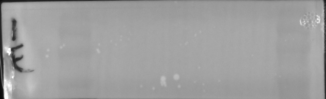


40

35

25

PAK2 58 kDa

GAPDH 36 kDa

Repeat 2

130

100

55

70

180

siCon

siNC

siPAK2-1

siPAK2-2


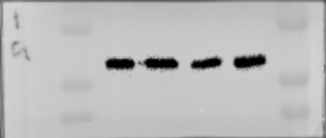

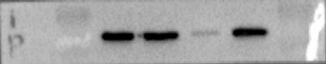


40

35

25

PAK2 58 kDa

GAPDH 36 kDa

Repeat 1

130

100

55

70

180


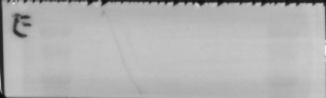

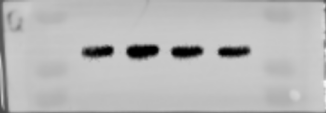

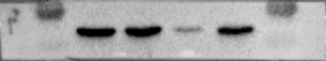


siCon

siNC

siPAK2-1

siPAK2-2

40

35

25

PAK2 58 kDa

GAPDH 36 kDa

Repeat 3

130

100

55

70

180

siCon

siNC

siPAK2-1

siPAK2-2


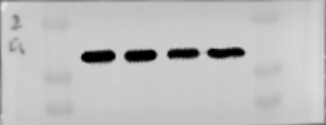

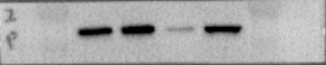

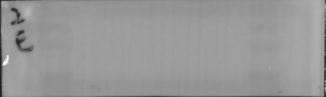


Figure 6c

40

35

25

PAK2 58 kDa

GAPDH 36 kDa

Repeat 2

150

100

50

70

100

150

E-cadherin 130 kDa

p-E-cadherin 130 kDa

Con

OE-PAK2


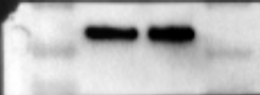

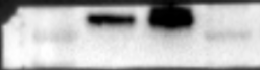

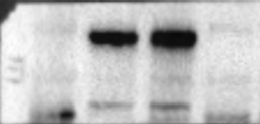

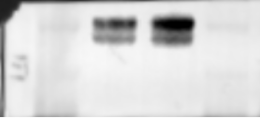


40

35

25

PAK2 58 kDa

GAPDH 36 kDa

Repeat 1

150

100

50

70

100

150

E-Cadherin

130 kDa

p-E-cadherin

130 kDa

Con

OE-PAK2


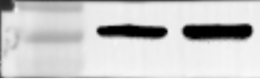

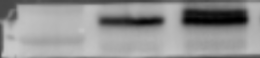

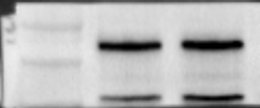

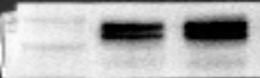


40

35

25

PAK2 58 kDa

GAPDH 36 kDa

Repeat 3

150

100

50

70

100

150

E-cadherin 130 kDa

p-E-cadherin 130 kDa

OE-PAK2

Con


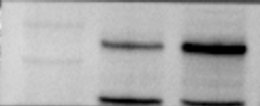


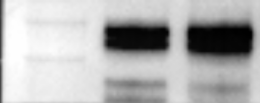


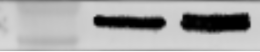

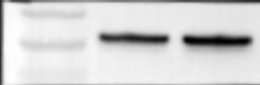


Figure 6e


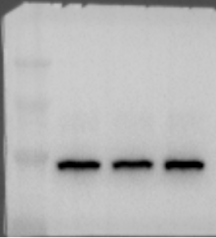

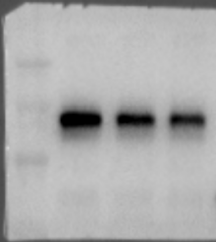

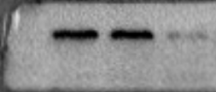

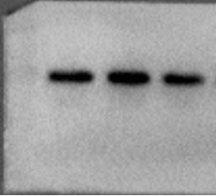


40

35

25

PAK2 58 kDa

GAPDH 36 kDa

Repeat 2

150

100

50

70

100

150

E-cadherin 130 kDa

p-E-cadherin 130 kDa

250

250

siCon

siPAK2-1


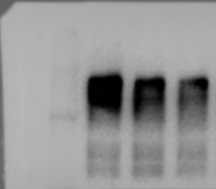

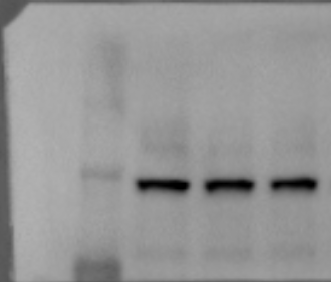

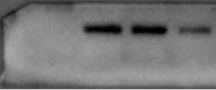

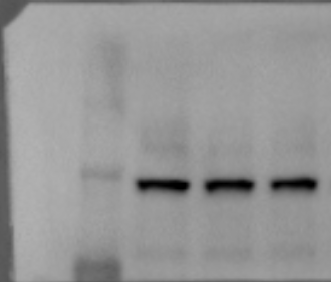


40

35

25

PAK2 58 kDa

GAPDH 36 kDa

Repeat 3

150

100

50

70

100

150

E-cadherin 130 kDa

p-E-cadherin 130 kDa

250

250

siCon

siPAK2-1

40

35

25

PAK2 58 kDa

GAPDH 36 kDa

Repeat 1

150

100

50

70

100

150

E-cadherin 130 kDa

p-E-cadherin 130 kDa

250

250

100

siCon

siPAK2-1


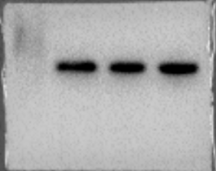

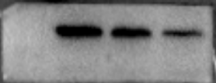

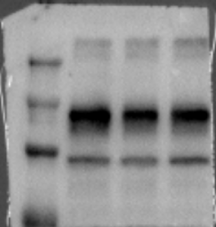

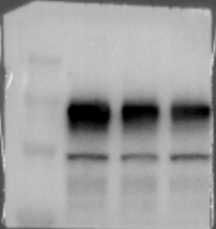


Figure 7


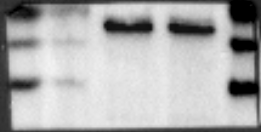

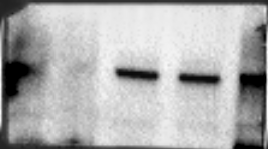

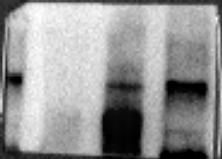

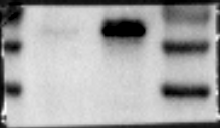


Input

Input

Control IgG

IP

150

100

70

55

40

E-cadherin 130 kDa

PAK2

58 kDa

Repeat 1


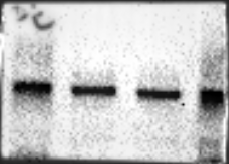

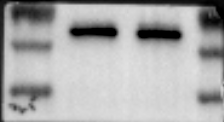

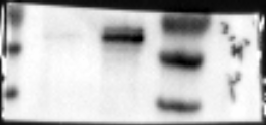

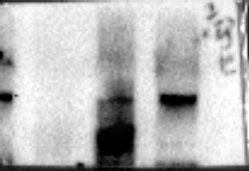


Input

Input

Control IgG

IP

150

100

70

55

40

E-cadherin 130 kDa

PAK2

58 kDa

Repeat 2


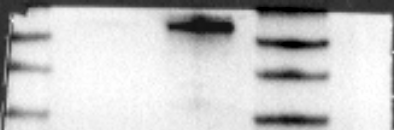

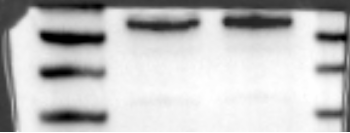

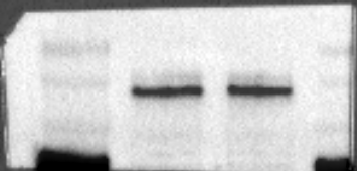

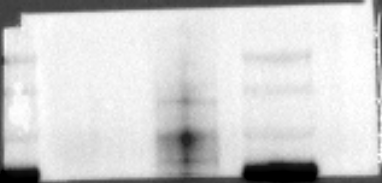


Input

Input

Control IgG

IP

E-cadherin 130 kDa

PAK2 58 kDa

150

100

70

55

40

35

Repeat 3

250

Figure 8b

70

55

40

35

25

PAK2 58 kDa

p-PAK2 58kDa

GAPDH 36 kDa

Repeat 1

55

70

Con

2.5um

5um

2.5um

5um

24h

48h


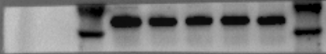

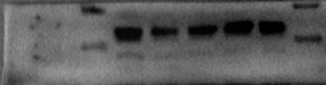

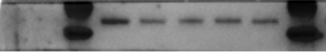


70

55

40

35

25

PAK2 58 kDa

p-PAK2 58kDa

GAPDH 36 kDa

Repeat 2

55

70


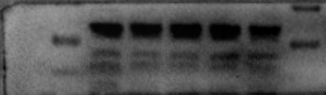

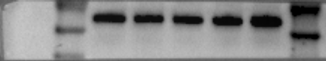

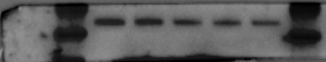


Con

2.5um

5um

2.5um

5um

24h

48h

70

55

40

35

25

PAK2 58 kDa

p-PAK2 58kDa

GAPDH 36 kDa

Repeat 3

55

70

Con

2.5um

5um

2.5um

5um

24h

48h


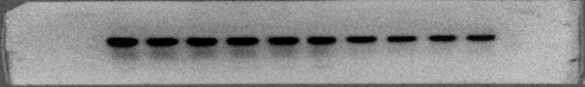

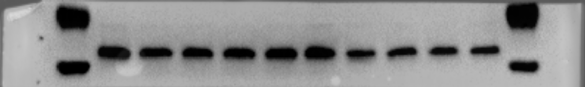

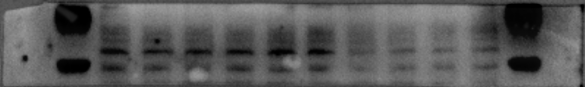


Figure 8d

5um


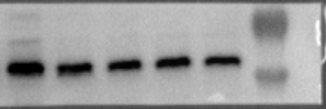

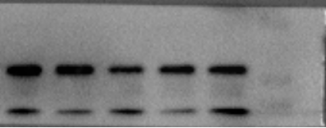

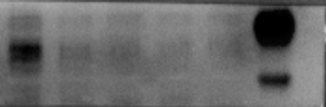

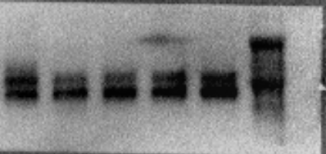

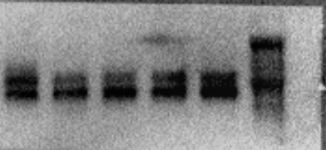


GAPDH 36 kDa

PAK2 58 kDa

35

40

55

70

70

55

130

180

180

130

Con

2.5um

p-PAK2 58 kDa

E-cadherin

130 kDa

p-E-cadherin

130 kDa

100

100

Repeat 2


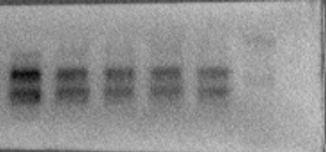

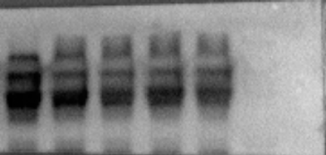

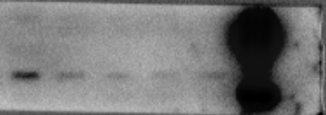

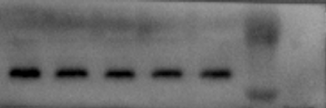

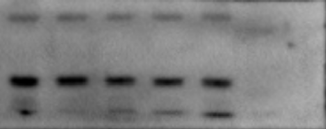


5um

GAPDH 36 kDa

PAK2 58 kDa

35

40

55

70

70

55

130

180

180

130

Con

2.5um

p-PAK2 58 kDa

E-cadherin

130 kDa

p-E-cadherin

130 kDa

100

100

Repeat 1


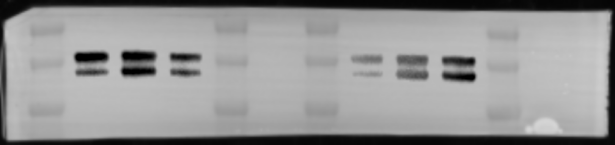

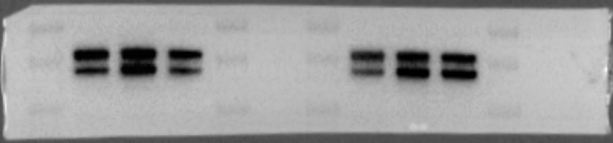

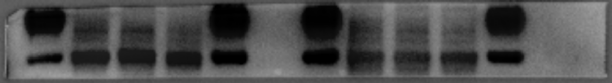

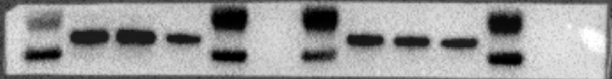

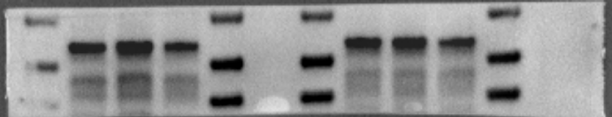


5um

GAPDH 36 kDa

PAK2 58 kDa

35

40

55

70

70

55

130

180

180

130

Con

2.5um

p-PAK2 58 kDa

E-cadherin

130 kDa

p-E-cadherin

130 kDa

100

100

Repeat 3
